# Prdx4 is a compartment-specific H_2_O_2_ sensor that regulates neurogenesis by controlling surface expression of GDE2

**DOI:** 10.1038/ncomms8006

**Published:** 2015-05-06

**Authors:** Ye Yan, Cynthia Wladyka, Junichi Fujii, Shanthini Sockanathan

**Affiliations:** 1 The Solomon Snyder Department of Neuroscience, The Johns Hopkins University School of Medicine, PCTB1004, 725 N Wolfe Street, Baltimore, Maryland 21205, USA; 2 Department of Biochemistry and Molecular Biology, Yamagata University, 2-2-2 Iidanishi, Yamagata 990-9585, Japan

## Abstract

Neural progenitors and terminally differentiated neurons show distinct redox profiles, suggesting that coupled-redox cascades regulate the initiation and progression of neuronal differentiation. Discrete cellular compartments have different redox environments and how they contribute to differentiation is unclear. Here we show that Prdx4, an endoplasmic reticulum (ER) enzyme that metabolizes H_2_O_2_, acts as a tunable regulator of neurogenesis via its compartmentalized thiol-oxidative function. Prdx4 ablation causes premature motor neuron differentiation and progenitor depletion, leading to imbalances in subtype-specific motor neurons. GDE2, a six-transmembrane protein that induces differentiation by downregulating Notch signalling through surface cleavage of GPI-anchored proteins, is targeted by Prdx4 oxidative activity. Prdx4 dimers generated by H_2_O_2_ metabolism oxidize two cysteine residues within the GDE2 enzymatic domain, which blocks GDE2 trafficking to the plasma membrane and prevents GDE2 neurogeneic function. Thus, Prdx4 oxidative activity acts as a sensor to directly couple neuronal differentiation with redox environments in the ER.

## Abstract (web summary)

Neuron differentiation is marked by changes in intracellular redox status. Here Yan *et al.* show that ER-resident peroxiredoxin 4 senses increased H_2_O_2_ and prevents the surface expression of differentiation-promoting GDE2 by modifying cysteine residues within GDE2.

## Graphical abstract

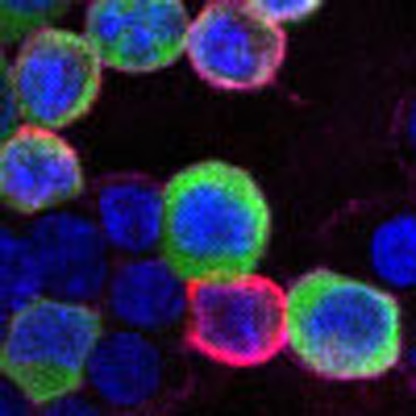

Cellular H_2_O_2_ levels in the nervous system show marked correlations with cellular proliferative states, with higher oxidative conditions in cycling progenitors compared with differentiated neurons^1^,^2^. Subtle redox changes can alter the thiol-redox states of proteins to regulate their function, and redox-dependent effects are known to impact fundamental cellular functions such as signal transduction, DNA and RNA synthesis, protein synthesis and cell cycle regulation^3^,^4^,^5^,^6^. These observations have led to the proposal that redox-coupled molecular cascades act as a series of ‘nano-switches' to regulate the timing and progression of neuronal differentiation^3^. Distinct cellular compartments such as the cytosol, nucleus, mitochondria and the endoplasmic reticulum (ER) have different redox environments^6^; thus, such a model would require that compartment-specific redox events individually or collectively synergize to control the transition from progenitor proliferation to neuronal differentiation. However, the identities of such redox pathways and how they might regulate the progression of neuronal differentiation are unclear.

Here, we identify the antioxidant enzyme Prdx4 as a tunable H_2_O_2_ sensor in the lumen of the ER that controls the timing of neurogenesis. Prdx4 is a 2-cysteine peroxiredoxin that is a major component of the ER oxidative protein folding pathway^7^,^8^,^9^,^10^. It metabolizes and removes H_2_O_2_ by-products generated by enzymes such as protein disulfide isomerase (PDI) and Ero1 during disulfide bond formation. After H_2_O_2_ removal, resultant Prdx4 dimers oxidize PDIs, which participate in the oxidative folding of new client proteins^9^,^10^,^11^. In times of oxidative stress, Prdx4 redox activity can target proteins such as the G-CSF (granulocyte-colony stimulating factor) receptor or the thromboxane A_2_ receptor for degradation^12^,^13^. Of note, Prdx4 reactive cysteines are highly susceptible to overoxidation, and accordingly, Prdx4 is inactive when H_2_O_2_ levels are high^14^,^15^. Here we identify a new function for Prdx4 thiol-oxidative activity in controlling the surface expression of GDE2, a six-transmembrane glycosylphosphatidylinositol (GPI) anchor-cleaving enzyme, with prominent roles in regulating neuronal differentiation^16^,^17^,^18^,^19^,^20^. Oxidized Prdx4 dimers prevent surface trafficking of GDE2 by oxidizing two cysteine residues within the GDE2 enzymatic domain, thereby abolishing its neurogeneic function. Prdx4 thus functions as a compartmentalized redox sensor that controls the timing of neurogenesis by coupling GDE2 surface expression in response to redox environments in the ER.

## Results

### Prdx4 expression in the developing spinal cord

**Figure 1:**
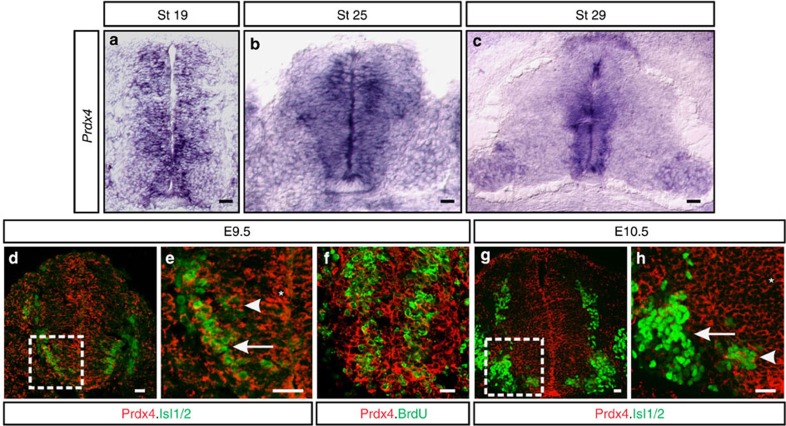
Prdx4 expression in the developing spinal cord. (**a–c**) *In situ* hybridization of transverse sections of embryonic chick spinal cords shows *Prdx4* transcript distribution. (**d**–**h**) Confocal micrographs of mouse spinal cords show the distribution of Prdx4 protein (red.) Boxes in **d** and **g** are magnified in **e** and **h**. Prdx4 is expressed in progenitors (*) and in newly differentiating (arrowheads), and postmitotic motor neurons (arrow in **e**); however Prdx4 is downregulated in terminally differentiated motor neurons at the end of neurogenesis (arrow in **h**). Scale bars, 20 μm.

In the developing spinal cord, cycling progenitors are distinguished from terminally differentiated neurons by cell-body position and molecular marker expression (Supplementary Fig. 1; refs 16, 21, 22). *Prdx4* transcripts were detected in cycling progenitors localized within the medial ventricular zone (VZ) and in newly differentiating neurons positioned more laterally within the intermediate zone (IZ) of the chick spinal cord (Fig. 1a–c). Similar distribution of Prdx4 protein was found in the mouse: Prdx4 is expressed in cycling motor neuron progenitors within the motor neuron progenitor domain (pMN) and in newly differentiating Isl1/2^+^ motor neurons (Fig. 1d,e). At E10.5, Prdx4 continues to be expressed in VZ and IZ cells, but is not expressed by fully differentiated Isl1/2^+^ motor neurons in the lateral marginal zone (Fig. 1g,h).

Physiological differences in redox can be detected by ARE (antioxidant response element)-luciferase reporters, which express luciferase in oxidative conditions via the binding and activity of the transcription factor Nrf2 (ref. 23). Electroporation of ARE-luciferase into chick spinal cords showed that Olig2^+^ progenitors in the VZ coexpressed luciferase as did a small number of cells in the IZ that express NeuroM, a transcription factor transiently expressed in newly differentiating neurons (Supplementary Fig. 1; ref. 24). Postmitotic neurons located in the marginal zone did not express luciferase. Thus, Prdx4 is expressed in oxidative zones in the spinal cord, and these correspond to VZ progenitors and newly differentiating neurons in the IZ.

### Prdx4 loss induces premature differentiation

**Figure 2:**
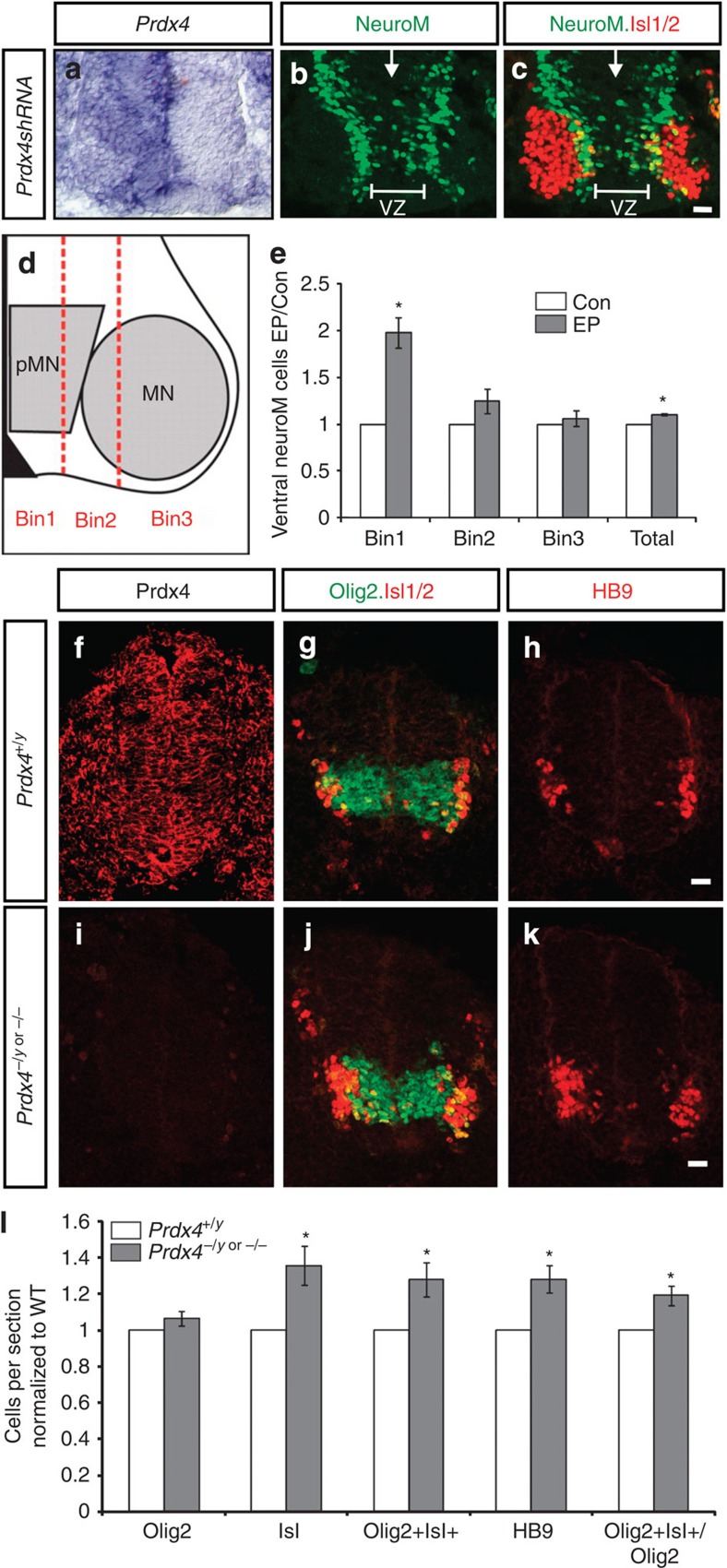
Prdx4 ablation triggers early initiation of neurogenesis. (**a**–**c**) Close-up of ventral embryonic chick spinal cords electroporated on the right side with *Prdx4* shRNAs. (**a**) *In situ* hybridization showing effective knockdown of *Prdx4* transcripts. (**b**,**c**) Antibody stains showing increased NeuroM and decreased Isl1/2^+^ motor neurons in the absence of Prdx4. VZ, ventricular zone; arrows mark midline. (**d**) Schematic shows division of lower right quadrant of spinal cord. pMN, motor neuron progenitor domain; MN, motor neurons. (**e**) Graph shows ratio of NeuroM^+^ cells in *Prdx4* shRNA electroporated (EP) spinal cords compared with the contralateral side (Con). Mean±s.e.m., *n*=5 embryos; Bin 1 *P=0.0039; Bin 2 *P*=0.1336; Bin 3 *P*=0.5132; total *P=0.0005, two-tailed Student's *t*-test. (**f**–**k**) Confocal images of transverse sections of E9.5 mouse spinal cords. (**l**) Graph shows ratio of cells in *Prdx4* knockout (KO) embryos compared with WT. Mean±s.e.m., *n*=4–9 embryos; Olig2^+^ *P*=0.1354; Isl1^+^ *P=0.0109; Olig2^+^Isl^+^ *P=0.0189; HB9^+^ *P=0.0059; Olig2^+^Isl^+^/Olig2^+^ *P=0.0069, two-tailed Student's *t*-test. Scale bars, 20 μm.

To investigate the physiological function of Prdx4, we ablated *Prdx4* expression by electroporating plasmids expressing short hairpin RNAs (shRNAs) engineered against the Prdx4 3′UTR (untranslated region) and coding sequence into chick spinal cords before motor neuron differentiation (Fig. 2a). Olig2 is expressed at high levels by motor neuron progenitors but is decreased in newly differentiating cells in the IZ. Spinal cords lacking *Prdx4* showed no changes in total numbers of Olig2^+^ cells; however, Olig2 expression was decreased in subsets of progenitors within the pMN, indicative of premature differentiation (Supplementary Fig. 2a–d). Consistent with this, *Prdx4* shRNA embryos showed a marked increase in cells expressing NeuroM (Fig. 2b–e), and additional NeuroM^+^ cells were located primarily in the VZ of *Prdx4* shRNA embryos (Bin 1; Fig. 2d,e). The increase in newly differentiating cells did not expand total Isl1/2^+^ motor neuron numbers but resulted in elevated cell death and a net reduction in motor neurons (Fig. 2c and Supplementary Fig. 2b,d,e). Mouse embryos lacking Prdx4 showed similar phenotypes (Fig. 2f,i; *Prdx4*^*−/y*^ or *Prdx4*^*−/−*^; ref. 25). Newly differentiating motor neurons in the IZ transiently coexpress Olig2 and Isl1/2 (refs 21, 22). *Prdx4* null embryos showed a roughly 30% increase of Olig2^+^Isl1/2^+^ cells (Fig. 2g,j,l) consistent with the premature onset of differentiation. In contrast to the chick, *Prdx4* null mutants exhibited a concomitant 20–40% increase in Isl1/2^+^ and HB9^+^ motor neurons (Fig. 2f–l), and no changes in cell death were detected between *Prdx4* mutants and wild-type (WT) animals (Supplementary Fig. 2f,g). These observations suggest that motor neuron progenitors lacking Prdx4 differentiate into postmitotic motor neurons ahead of schedule.

### Prdx4 loss alters cell cycle exit and motor neuron identity

**Figure 3:**
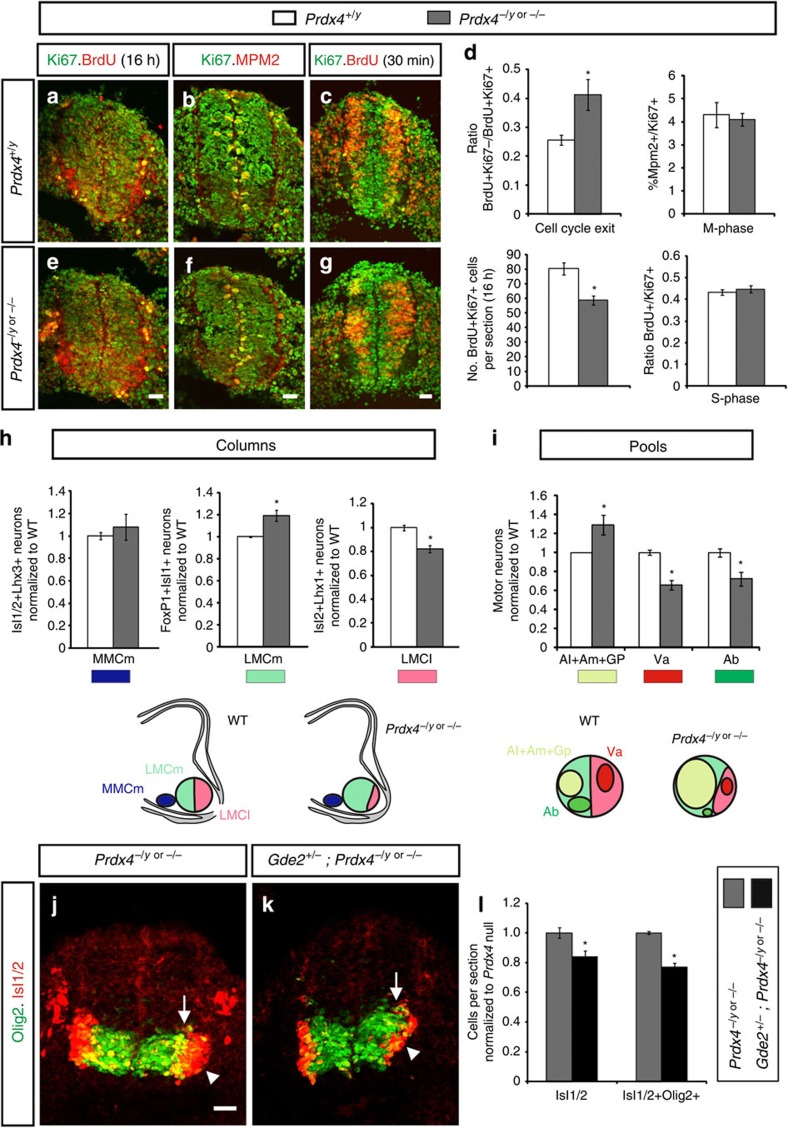
Prdx4 genetically interacts with GDE2 to control the timing of neurogenesis. (**a**–**c**,**e**–**g**) Sections of mouse embryonic spinal cords stained for cell cycle analysis. BrdU labelling was performed 16 h (**a**,**e**) or 30 min before analysis (**c**,**g**). (**d**) Graphs quantifying different phases of the cell cycle. Mean±s.e.m., *n*=3–7 embryos; cell cycle exit *P=0.0321; M-phase *P*=0.7474; total number of BrdU^+^Ki67^+^ cells (16 h) *P=0.0111; S-phase *P*=0.4660, two-tailed Student's *t*-test. (**h**) Graphs quantifying ratios of motor columns compared with WT animals. Mean±s.e.m., *n*=4–5 embryos; medial median motor column (MMCm) *P*=0.5395; medial divisions of lateral motor column (LMCm) *P=0.0154; lateral divisions of lateral motor column (LMCl) *P=0.0025; two-tailed Student's *t*-test; lower panels are schematics showing changes in motor columns in the absence of Prdx4. (**i**) Graphs quantifying ratios of motor pools compared with WT animals. Mean±s.e.m., *n*=5–6 embryos; Al (adductor longus)+Am (adductor magnus)+Gp (gracilis posterior) *P=0.0385; Va (vasti) *P=0.0003; Ab (adductor brevis) *P=0.0159; two-tailed Student's *t*-test; lower panels are schematics showing changes in motor pools in Prdx4 KOs. (**j**,**k**) Sections of E9.5 mouse spinal cords. Arrows mark newly differentiating Olig2^+^Isl^+^ cells; arrowheads mark postmitotic Isl motor neurons. (**l**) Graphs quantifying ratios of Olig2^+^Isl^+^ cells and Isl^+^ cells compared with *Prdx4* mutant animals. Mean±s.e.m., *n*=6–8 embryos; Isl^+^ *P=0.01; Olig2^+^Isl^+^ *P=0.0001; two-tailed Student's *t*-test. Scale bars, 20 μm.

To determine how the loss of Prdx4 affects cell cycle progression, we performed pulse labelling studies using the nucleotide analogue 5-bromodeoxyuridine (BrdU) in *Prdx4* null mice and WT littermates. No changes in the proportion of cells in S-phase after a 30-min BrdU pulse (BrdU^+^/Ki67^+^; Ki67 marks all cycling cells; Fig. 3c,d,g) or in M-phase (Mpm2^+^/Ki67^+^; Fig. 3b,d,f) were observed between *Prdx4* nulls and controls, implying similar cell cycle lengths in both groups (Fig. 3d). However, the total number of BrdU^+^Ki67^+^ cells in *Prdx4* mutants was reduced by 27% compared with WT siblings when a 16-h BrdU pulse was administered (Fig. 3a,d,e). This observation suggests that the overall number of cycling cells is decreased when *Prdx4* is ablated. In addition, cell cycle exit indices (BrdU^+^Ki67^−^: BrdU^+^Ki67^+^) were increased by ∼60% in *Prdx4* null animals compared with WT littermates (Fig. 3a,d,e). These collective observations indicate that Prdx4 ablation forces premature cell cycle exit of progenitors resulting in increased postmitotic motor neuron numbers.

Time of cell cycle exit is linked to the acquisition of specific motor neuron fates, thus loss of Prdx4 could cause imbalances in motor columns and pools. We compared the numbers of medial median motor column (MMCm) and medial and lateral divisions of lateral motor column (LMC) neurons at *Prdx4* null and WT hindlimb level spinal cords using distinguishing molecular markers (Supplementary Fig. 3a–f,k; refs 21, 22). No changes in MMCm motor column motor neuron numbers were detected between *Prdx4* nulls and WT littermates (Fig. 3h and Supplementary Fig. 3a,d). However, early-born medial LMC motor neurons were increased by 20% with a concomitant decrease in late-born lateral LMC neurons (Fig. 3h and Supplementary Fig. 3b–f). Motor neurons within the medial and lateral LMC that project to the same muscles are clustered into motor pools and are molecularly distinct (Supplementary Fig. 3g–k). Consistent with increases in early-born motor neuron subtype populations, *Prdx4* mutants showed an increase in early-born adductor longus, adductor magnus and gracilis posterior motor pools and a corresponding decrease in late-born adductor brevis and vasti motor pools (Fig. 3i and Supplementary Fig. 3g–k; refs 18, 26).

### Prdx4 and GDE2 interact to regulate neurogenesis

What is the mechanism that underlies Prdx4 temporal control of neurogenesis? We noted that *Prdx4* null phenotypes are directly opposite to phenotypes exhibited by embryos lacking GDE2, a six-transmembrane protein that specifically regulates the timing of LMC motor neuron differentiation in the spinal cord^16^,^17^,^18^,^19^,^20^. GDE2 and Prdx4 are expressed in the IZ where newly differentiating motor neurons are found (Fig. 1; refs 16, 18). We hypothesized that *Prdx4* null animals have increased GDE2 activity, and that, normally, Prdx4 inhibits GDE2-dependent stimulation of neurogenesis. We therefore compared the extent of premature differentiation in *Prdx4* null mutants (*Prdx4*^*−/y*^ or *Prdx4*^*−/−*^) that lack one allele of *Gde2*. We found that the increase in newly differentiating Olig2^+^Isl^+^ cells and Isl1/2 motor neurons normally observed in *Prdx4* null animals was substantially reduced in *Prdx4*^*−/y*^*;Gde2*^*+/−*^ or *Prdx4*^*−/−*^*;Gde2*^*+/−*^ embryos (Fig. 3j–l). The suppression of premature differentiation in *Prdx4* nulls by genetic removal of one allele of *Gde2* suggests that Prdx4 negatively regulates GDE2 activity to control the timing of neurogenesis.

### Prdx4 thiol-redox activity inhibits GDE2 function

**Figure 4:**
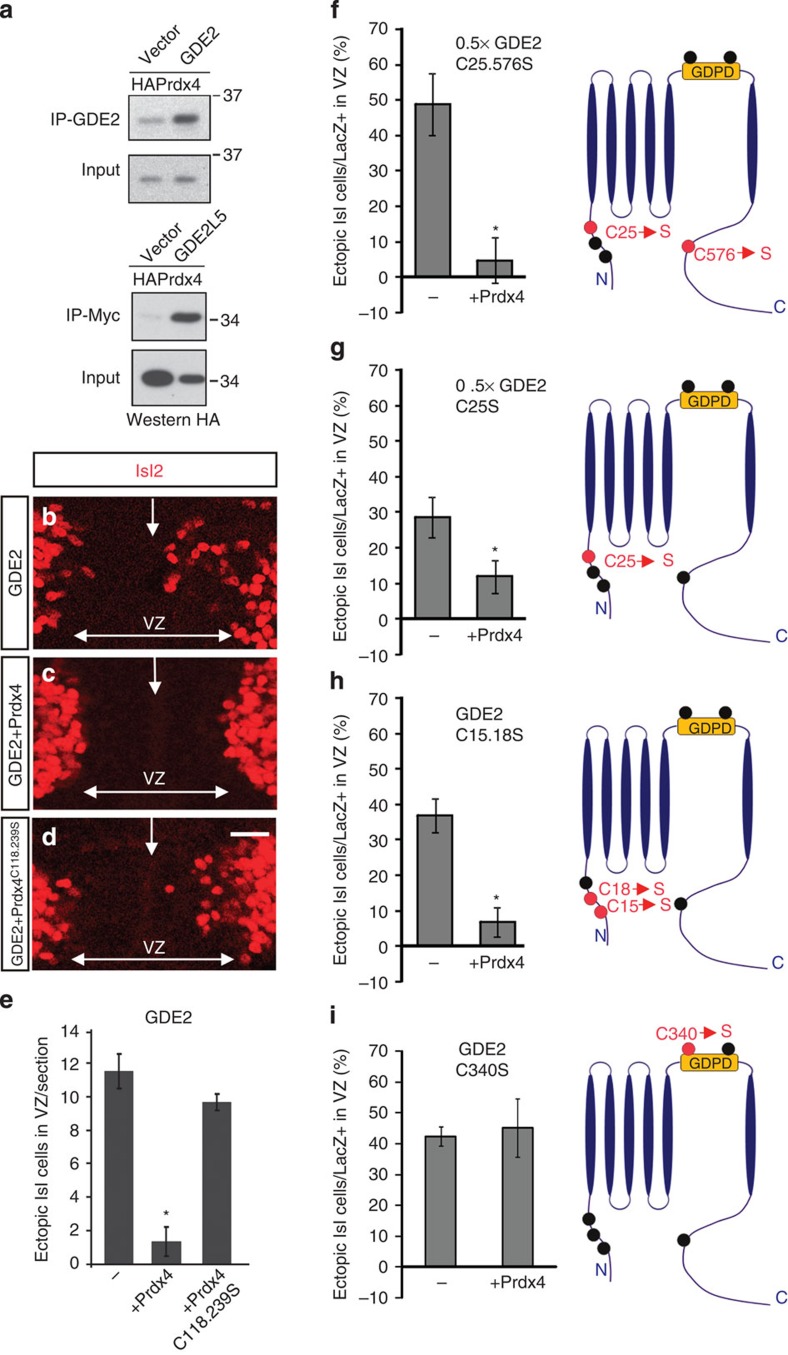
Prdx4 inhibits GDE2 activity by targeting its extracellular Cys residues. (**a**) Representative western blot (one of three individual experiments) shows co-IP of Prdx4 and GDE2 using extracts from transfected HEK293T cells. (**b**–**d**) Close-up of ventral regions of chick spinal cords that are electroporated on the right side. VZ, ventricular zone; arrows mark midline. Scale bars, 20 μm. Graphs quantifying number of Isl1/2^+^ cells (**e**) or percentage of LacZ^+^ cells that are Isl1/2^+^ (**f**–**i**) are induced in VZ progenitors. GDE2 expressing constructs were bicistronic for LacZ; LacZ was used as a measure of electroporation efficiency. Mean±s.e.m., schematics on the right show location of Cys residues (black circles) in GDE2, red circles are Cys residues that were mutated to Ser. 0.5 × GDE2 was used in **f** and **g** as these versions of GDE2 are hyperactive (**e**) GDE2+Prdx4 *P=3.7864E-05; GDE2+Prdx4C118.239S *P*=0.1519; *n*=4–7 embryos; (**f**) 0.5 × GDE2C25.576S+Prdx4 *P=0.0009, *n*=9–12 embryos; (**g**) 0.5 × GDE2C25S+Prdx4 *P=0.0425, *n*=5–10 embryos; (**h**) GDE2C15.18S+Prdx4 *P=0.0003, *n*=8–9 embryos; (**i**) GDE2C340S+Prdx4 *P*=0.7888, *n*=5–6 embryos, two-tailed Student's *t*-test.

Co-immunoprecipitation (co-IP) assays using extracts prepared from transfected HEK293T cells showed that Prdx4 and GDE2 are capable of forming protein complexes (Fig. 4a). Thus, Prdx4 regulation of GDE2 activity is likely to be direct. To test whether Prdx4 inhibits GDE2 activity, we utilized established *in vivo* assays of GDE2 function in electroporated chick embryos^16^,^17^. Unilateral GDE2 overexpression in embryonic chick spinal cords triggers premature differentiation manifest by the formation of many ectopic Isl2^+^ motor neurons in the electroporated VZ but not in the contralateral unelectroporated VZ (Fig. 4b,e). Strikingly, coexpression of Prdx4 with GDE2 suppressed the ability of GDE2 to induce Isl2^+^ motor neuron differentiation (Fig. 4c,e). Coexpression of GDE2 and a redox inactive version of Prdx4 that substitutes its reactive cysteine residues with serine (Prdx4C118.239S) resulted in many ectopic Isl2^+^ motor neurons, equivalent to when GDE2 is overexpressed alone (Fig. 4d,e; refs 9, 27, 28). Co-IP assays confirmed that Prdx4C118.239S retained the ability to interact with GDE2 (Supplementary Fig. 4b). These observations suggest that Prdx4 interacts with GDE2 and utilizes its thiol-redox activity to inhibit GDE2-dependent induction of motor neuron differentiation.

To define the sites of Prdx4 redox activity on GDE2, we mutated individual and paired cysteine (C) residues within GDE2 to serine (S). With the exception of GDE2 carrying a double mutation at C340 and C467 (GDE2C340.467S), all GDE2 variants showed appropriate glycosylation and surface expression, and maintained their abilities to interact with Prdx4 in co-IP assays (Supplementary Fig. 4a–c). GDE2C25.576S, GDE2C25S, GDE2C15.18S, GDE2C340S and GDE2C467S readily induced motor neuron differentiation in the VZ of electroporated embryonic chick spinal cords (Fig. 4f–i; data not shown). Prdx4 coexpression with the GDE2 variants effectively blocked GDE2C25.576S, GDE2C25S and GDE2C15.18S activity (Fig. 4f–h). However, Prdx4 failed to inhibit the inductive activities of GDE2C340S or GDE2C467S, suggesting that the thiol residues located within the extracellular GDPD domain are the sites of Prdx4 inhibitory redox activity (Fig. 4i; data not shown). Consistent with this observation, co-IP assays demonstrate that Prdx4 can interact with GDE2 via the GDPD region where C340 and C476 are located (Fig. 4a).

### Prdx4 inhibits GDE2 through thiol oxidation

**Figure 5:**
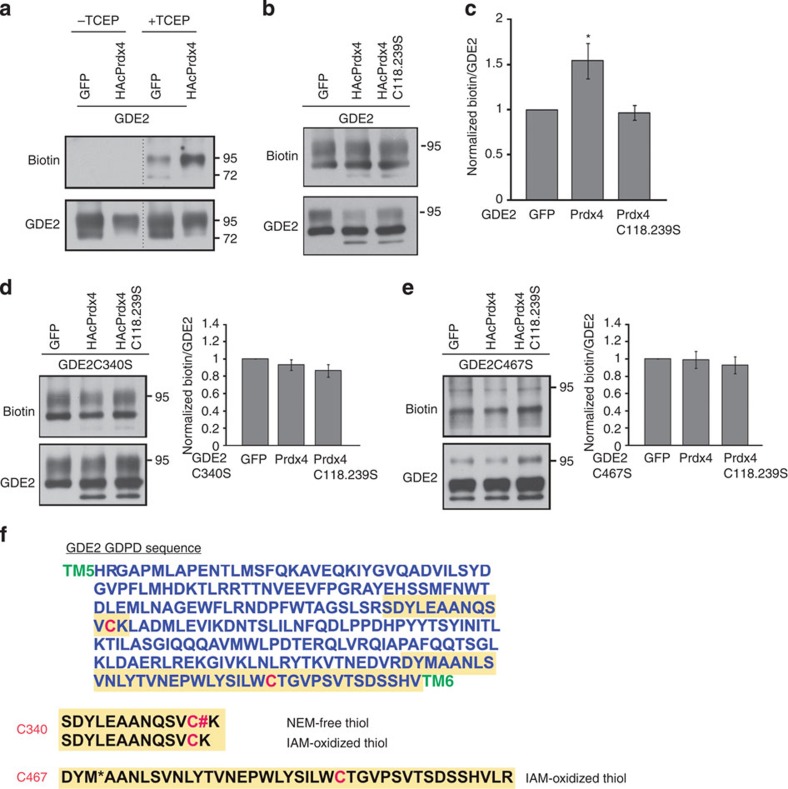
Prdx4 oxidizes Cys residues within the GDE2 GDPD domain. (**a**,**b**,**d**,**e**) Western blots of biotin labelling assays to measure thiol oxidation. (**c**–**e**) Graph quantifying biotin incorporation normalized to total GDE2, GDE2C340S or GDE2C467S. Mean±s.e.m., *n*=8–13 different transfection experiments. Compared with GFP (green fluorescent protein) controls, (**c**) Prdx4 *P=0.0159, Prdx4C118.239S *P*=0.6955; (**d**) Prdx4 *P*=0.3022, Prdx4C118.239S *P*=0.0854; (**e**) Prdx4 *P*=0.9249, Prdx4C118.239S *P*=0.4988, two-tailed Student's *t*-test. (**f**) Sequence of the GDE2 GDPD domain located between transmembrane (TM) regions 5 and 6. Sequences in yellow denote peptides identified by mass spectrometry; C340 and C467 are shown in red.

To examine how Prdx4 alters thiol-redox states of GDE2, we utilized biotin-labelling assays to quantify the levels of oxidized thiol groups in GDE2 in the absence or presence of Prdx4 in HEK293T cell extracts^17^. Free thiol groups were first blocked by addition of N-ethylmaleimide (NEM) to cell extracts, and existing disulfide bonds were reduced by tris (2-carboxyethyl) phosphine (TCEP). Resultant free thiols were labelled with Biotin-HPDP (N-[6-(Biotinamido)hexyl]-3-(2-pyridyldithio)propionamide) and quantified by densitometric analysis of IP-ed (immunoprecipitated) GDE2 on western blots. This method effectively blocked free thiol groups in GDE2 as IP-ed GDE2 was not labelled by biotin in the absence of the reducing agent TCEP (Fig. 5a). Addition of TCEP led to GDE2 biotinylation consistent with earlier reports that GDE2 contains endogenous disulfide bonds (Fig. 5a; ref. 17). Coexpression of Prdx4 with GDE2 increased levels of biotinylated GDE2, suggesting that Prdx4 oxidizes GDE2 (Fig. 5a). The extent of biotin increase is variable and likely reflects the redox sensitivity of the extracellular GDE2 Cys residues (Fig. 5a–c). Nonetheless, when redox inactive Prdx4C118.239S was coexpressed with GDE2, no quantitative changes in GDE2 biotinylation were observed (Fig. 5b,c). We repeated the biotin-labelling experiments, this time coexpressing Prdx4 with either GDE2C340S or GDE2C467S. The amounts of biotinylated GDE2C340S and GDE2C467S remained similar whether Prdx4 was present or absent, which is in agreement with the extracellular cysteine residues being the sites of Prdx4 oxidative function (Fig. 5d,e). Tandem liquid chromatography-tandem mass spectrometry (LC-MS/MS) analysis of GDE2 in HEK293T cells confirmed that the extracellular C340 and C467 residues are capable of being oxidized^17^. Here, free thiol groups were blocked by incubation with NEM, existing oxidized thiols were reduced with dithiothreitol and newly reduced thiols were modified with iodoacetamide (IAM). LC-MS/MS analysis of IP-ed GDE2 showed that peptides containing C340 and C467 were labelled by NEM and IAM, respectively, while LC-MS analysis identified peptides containing C340 labelled with IAM (Fig. 5f). These collective observations suggest that Prdx4 inhibits GDE2 activity by oxidation of the C340 and C467 residues located in the GDE2 extracellular enzymatic domain.

### Prdx4 inhibits GDE2 surface trafficking

**Figure 6:**
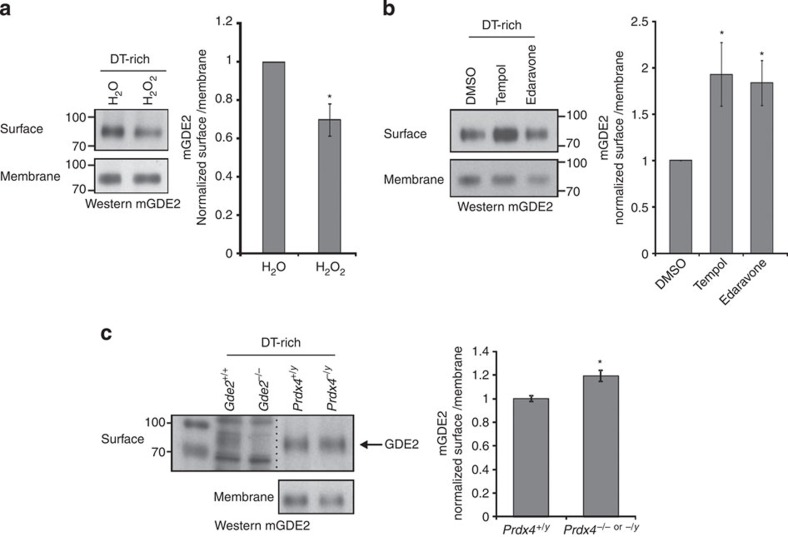
GDE2 surface expression in motor neurons is redox sensitive and Prdx4 dependent. (**a–c**) Western blots of surface biotinylation assays using membrane fractions of primary motor neuron cultures derived from E11.5 spinal cords. In **c**, arrow marks detection of band corresponding to GDE2, which is absent in *Gde2* null animals. Graphs show surface levels of biotinylated GDE2 normalized to total levels of membrane GDE2. Mean±s.e.m., (**a**) *P=0.003, *n*=14 separate cultures; (**b**) Tempol *P=0.021, Edaravone *P=0.006, *n*=11 separate cultures; (**c**) *P=0.035, *n*=9–24 embryos; two-tailed Student's *t*-test.

We confirmed that Prdx4 is localized to the ER in motor neurons, as Prdx4 colocalized with the ER marker KDEL in sectioned mouse spinal cords and in primary motor neuron cultures (Supplementary Fig. 5; refs 29, 30). Moreover, Triton X-114 extraction of transfected HEK293T cells consistently detected mPrdx4 in the detergent-free (DT-free) hydrophilic phase where cytoplasmic and luminal proteins accumulate and not in the detergent-rich (DT-rich) membrane fraction (Supplementary Fig. 5). Membrane-bound proteins are normally trafficked to the cell surface from the ER, raising the possibility that Prdx4 oxidizes GDE2 and prevents its transport to the plasma membrane. If so, then GDE2 surface expression should be redox sensitive. Treatment of primary motor neuron cultures with H_2_O_2_ significantly decreased levels of surface biotinylated GDE2, whereas incubation of cultures with established reactive oxygen species (ROS) scavengers, Tempol and Edaravone, elevated GDE2 surface expression (Fig. 6a,b; ref. 31). Consistent with changes in cellular oxidative states upon addition of these compounds, Prdx4 dimer to monomer ratios were increased upon H_2_O_2_ addition and decreased when incubated with Tempol and Edaravone (Supplementary Fig. 6). The altered ratios of Prdx4 dimers:monomers further indicate that the levels of oxidized Prdx4 dimers capable of oxidizing GDE2 were increased by H_2_O_2_ treatment in motor neuron cultures and decreased by ROS scavengers (Supplementary Fig. 6). Taken together, higher oxidative conditions decrease surface levels of GDE2 in motor neurons while less-oxidized conditions stimulate GDE2 surface expression. Moreover, the changes in GDE2 surface localization are inversely correlated with Prdx4 oxidative states. To test the physiological consequence of Prdx4 activity on GDE2 surface expression, we compared the levels of surface biotinylated GDE2 protein in primary motor neuron cultures prepared from WT and *Prdx4* null spinal cords. Surface expression of GDE2 was increased by ∼20% in the absence of mPrdx4 (Fig. 6c). This degree of change in GDE2 surface expression is consistent with the restriction of Prdx4 and GDE2 coexpression to newly born motor neuron populations and the lack of Prdx4 expression in terminally differentiated motor neurons (Fig. 1).

**Figure 7:**
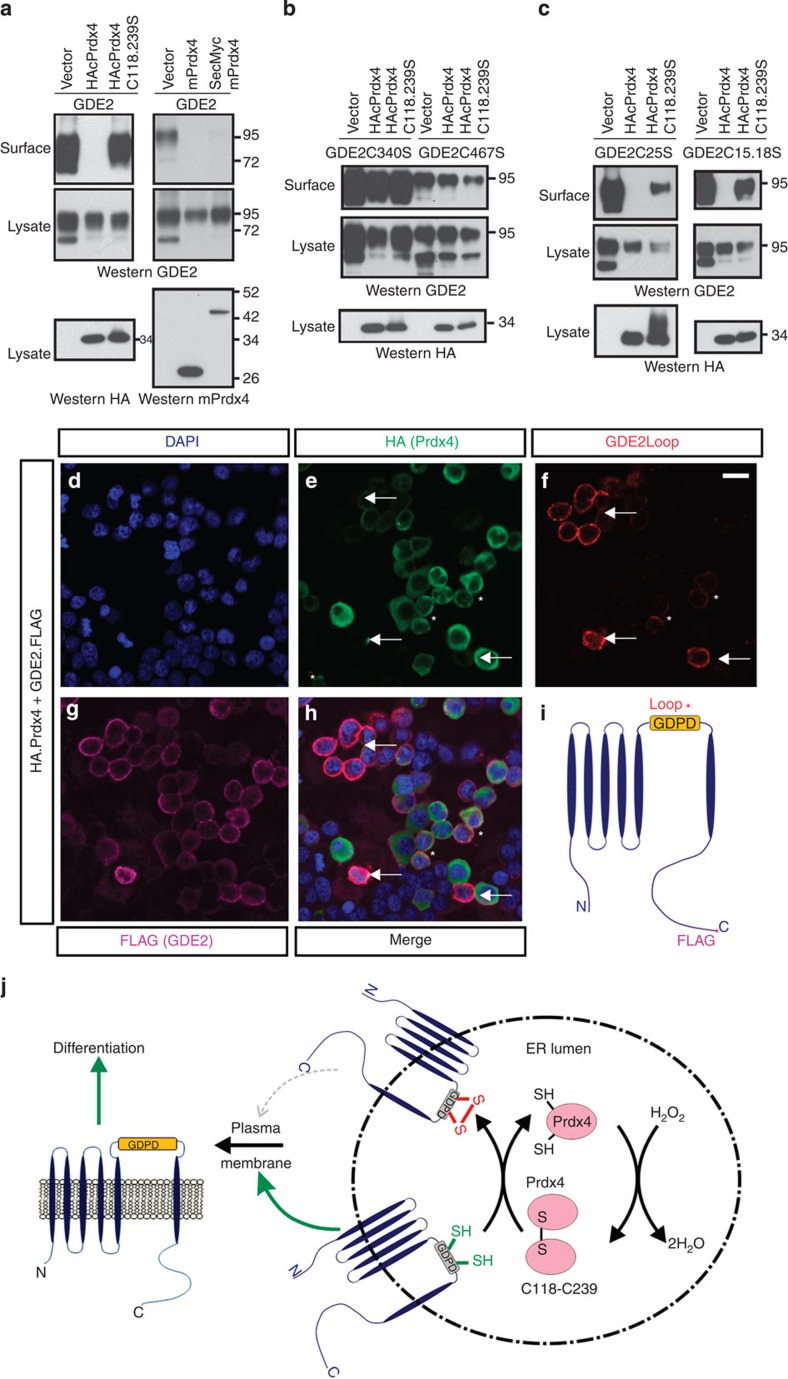
Prdx4 oxidative activity blocks GDE2 trafficking. (**a**–**c**) Western blots of surface biotinylation assays in transfected HEK293T cells. These are representative of (**a**) *n*=7, (**b**) *n*=6, (**c**) *n*=5–7 different transfection experiments. (**d**–**h**) Live-cell staining of Prdx4 (green), GDE2 (FLAG, purple) and surface GDE2 (red) in transfected HEK293T cells. Panels represent one of 20 different areas imaged for each experiment. Arrows mark cells with surface GDE2 expression; asterisks (*) marks cells with very low surface GDE2 expression. Scale bar, 20 μm. (**i**) Schematic diagram of GDE2 showing epitope locations for FLAG and GDE2Loop antibodies utilized in **d**–**h**. (**j**) Model of Prdx4 inhibition of GDE2 function. Prdx4 monomers in the ER lumen are oxidized by H_2_O_2_ at their redox active C118 and C239 Cys to form dimers; these dimers subsequently oxidize Cys residues in the GDE2 GDPD domain, which inhibits GDE2 surface localization. When Prdx4 dimer levels decrease, the GDE2 GDPD domain escapes oxidation, and GDE2 traffics to the cell surface where it induces neurogenesis of neighbouring progenitors by GPI-anchor cleavage.

To determine whether Prdx4 is sufficient to prevent GDE2 surface expression, we performed surface biotinylation assays in HEK293T cells cotransfected with GDE2 and tagged versions of Prdx4. Levels of surface biotinylated GDE2 were markedly reduced by Prdx4, consistent with our studies in motor neurons, but not by redox inactive Prdx4C118.239S (Fig. 7a). Similar results were obtained with mPrdx4 consistent with conserved functions of Prdx4 in mouse and chick. In accordance with our earlier analyses, Prdx4 failed to reduce the levels of surface biotinylated GDE2C340S and GDE2C467S, but effectively inhibited surface accumulation of GDE2C25S and GDE2C15.18S (Fig. 7b,c).

Prdx4 is localized to the ER in motor neurons but is also reported to be secreted^32^. We fused Prdx4 to a secretory signal that targets proteins to the ER lumen (SecMycmPrdx4) and confirmed its partitioning to DT-free fractions after Triton X-114 extraction of transfected HEK293T cells, consistent with localization of SecMycmPrdx4 to the ER lumen. SecMycmPrdx4 effectively reduced levels of biotinylated GDE2; reinforcing the model that Prdx4 inhibition of GDE2 occurs within the ER (Fig. 7a and Supplementary Fig. 5). If Prdx4 function occurs within the ER, we would expect Prdx4 suppression of GDE2 surface expression to be cell autonomous. Plasmids expressing human influenza hemagglutinin (HA) Prdx4 and GDE2.FLAG were transfected into HEK293T cells, and the distribution of GDE2 was compared with Prdx4. Live-cell staining to detect surface GDE2 using a polyclonal antibody (GDE2Loop) against the GDE2 extracellular enzymatic domain was performed before permeabilization and visualization of intracellular FLAG and HA epitopes on GDE2 and Prdx4, respectively (Fig. 7i). Many transfected cells coexpressed GDE2 (purple) and Prdx4 (green) as visualized by FLAG and HA immunoreactivity (Fig. 7d–h); however, the majority of HA^+^ FLAG^+^ cells did not express GDE2 on the cell surface (red, Fig. 7f). In rare cases, where GDE2 surface expression was detected in Prdx4^+^ cells, minor GDE2Loop immunoreactivity was detected, indicative of very low levels of GDE2 at the plasma membrane (asterisks, Fig. 7e,f,h). In contrast, cells that expressed GDE2 on the cell surface (GDE2Loop^+^) did not coexpress Prdx4 (arrows) even when adjacent to cells expressing high levels of Prdx4. These observations indicate that Prdx4 inhibition of GDE2 is cell autonomous, and reinforces the model that Prdx4 function in the ER inhibits surface expression of GDE2.

## Discussion

Prdx4 is the only peroxiredoxin located within the ER, where it metabolizes H_2_O_2_ and oxidizes PDIs that play central roles in oxidative protein folding^9^,^10^,^11^. Our study identifies a new role for Prdx4 oxidative properties in regulating the timing of neurogenesis through redox-dependent control of GDE2 surface expression (Fig. 7j). Prdx4 regulation of GDE2 trafficking is not due to altered folding of GDE2, as GDE2, GDE2C340S and GDE2C467S proteins are stable and normally glycosylated in the presence of Prdx4, and GDE2C340S and GDE2C467S exhibit appropriate surface localization and are physiologically active in terms of inducing motor neuron differentiation. Instead, H_2_O_2_ levels in the ER modulate the availability of Prdx4 dimers that can oxidize GDE2 and regulate its surface expression, enabling Prdx4 to serve as a finely tuned regulator of GDE2 function in response to subtle physiological changes in the ER redox environment. The susceptibility of GDE2 Cys residues to oxidation raises the question whether ROS can directly modify these Cys residues independently of Prdx4 to inhibit GDE2 surface localization. Arguing against this possibility is our observation that application of H_2_O_2_ to primary motor neuron cultures derived from *Prdx4* null mice does not decrease GDE2 surface expression (Supplementary Fig. 7). Instead, GDE2 surface expression is further increased under these conditions, suggesting that Prdx4 negatively regulates additional redox-dependent mechanisms in the ER that promote GDE2 surface localization. Taken together, these observations indicate that GDE2 trafficking is regulated through complex redox-dependent pathways in the ER, and suggest that Prdx4 occupies central roles in the redox-dependent control of GDE2 surface expression.

Prdx4 and GDE2 are coexpressed in newly differentiating neurons in the IZ. Our analysis of ARE-luciferase reporter expression shows that IZ cells exhibit mosaic luciferase expression, suggesting that newly differentiating neurons in the IZ display a range of redox levels as they transition towards a terminally differentiated state. These differences in IZ redox levels would vary surface levels of GDE2 within this region through Prdx4-dependent regulation of GDE2 trafficking. GDE2 surface expression is critical for its ability to induce differentiation; it utilizes its extracellular enzymatic function to cleave and inactivate the GPI-anchored protein RECK on the surface of IZ cells^20^. RECK inactivation releases inhibition of ADAM10 that stimulates shedding of the Notch ligand from the cell membrane. This in turn downregulates Notch signalling in neighbouring progenitors and induces differentiation^20^. Thus, attenuation of GDE2 surface expression by Prdx4 will significantly impact the ability of GDE2 to initiate progenitor differentiation. Importantly, GDE2 activity at the cell surface is also subject to redox control through Prdx4-independent mechanisms^17^. GDE2 cell surface activity is gated by an intracellular disulfide bond formed between Cys residues in the N- and C-terminal domains; this inhibition is released by the thiol-redox activity of Prdx1, a cytoplasmic 2-Cys peroxiredoxin related to Prdx4 that is expressed in IZ cells. Taking this into consideration, we propose a model where GDE2 activity is regulated in two different cellular compartments by opposing redox-dependent mechanisms; within the ER, Prdx4 dimers generated after metabolizing H_2_O_2_, oxidizes GDE2 within its enzymatic domain to inhibit its trafficking to the cell surface, whereas Prdx1 reduces an intracellular disulfide bond to activate GDE2 at the plasma membrane. Thus, redox-dependent regulation of GDE2 activity in discrete cellular compartments by Prdx proteins requires integration to ensure the appropriate temporal progression of neurogenesis. GDE3 and GDE6 are in the same family as GDE2 and share conserved cysteine residues^16^,^20^,^33^. Thus, compartmentalized thiol-oxidative regulation of six-transmembrane GDE surface expression by Prdx4 could constitute a key mechanism to attenuate GDE enzymatic control of GPI-anchored protein function.

## Methods

### Expression constructs

Chick Prdx4 ORF was PCR cloned using cDNA plasmid from University of Delaware (clone ID: pgm2n.pk011.C15) as template. The same sequence was also obtained by reverse transcription (RT)-PCR from HH St21–23 chicken spinal cord. The nucleotide sequence was identical to XM_416800.2 (updated to XM_416800.4) except two nucleotide difference (G139 to A and A487 to G), which did not change the protein sequence (same as NP_416800.2). Mouse Prdx4 ORF was PCR cloned using cDNA plasmid from Open Biosystem (Image ID: 3481827). All chick Prdx4 expression constructs were cloned in the chick β-actin promoter vector (pCAGGS) and CMV IE94 promoter vector (pCS2) with three HA tag on the N terminus unless otherwise specified. Mouse Prdx4 expression constructs were cloned in the pCAGGS vector with no tag or SecMyc tag (secretory signal with 6 myc) on the N terminus^20^. Chick GDE2 expression constructs were cloned in pCAGGS vector with no tag, one FLAG tag on the N terminus or three FLAG tag on C terminus^17^,^20^. GDE2 loop 5 (GDE2L5) was cloned in frame into the N terminus of the myc-tagged platelet-derived growth factor (PDGF) receptor transmembrane domain of pDisplay vector. All mutant constructs were generated by site-directed (Quickchange kit, Stratagene) or PCR-based mutagenesis.

### *In situ* hybridization and immunofluorescence

For immunostaining analyses, 12.5 μm sections were generated from fixed embryos and slides were air-dried for 20 min. Slides were blocked in PBT (PBS, 0.1% Triton X-100, 1% BSA) for 1 h at room temperature (RT) in a humidified chamber and then incubated in PBT with the relevant concentration of antibody overnight at 4 °C. Slides were washed in PBS (3 × 10 min) and incubated in PBT with secondary antibody for 40 min at RT and washed again (3 × 10 min PBS). Slides were mounted with coverslips in Vectashield (Vector Laboratories). For BrdU staining, slides were incubated in 600 ml of 10 mM sodium citrate, pH 6.0, and microwaved for 20 min, replacing evaporated water after 10 min. Slides were cooled for 20 min, washed 3 × 5 min in water followed by 1 × 5 min in PBS. Slides were processed for immunostaining as described above.

*In situ* hybridization analyses were performed as follows: slides were fixed in 4% paraformaldehyde in PBS for 10 min, washed in PBS (3 × 3 min) and digested in proteinase K (1 mg ml^−1^ in 50 mM Tris (pH 7.5) and 5 mM EDTA) for 5 min at RT. Slides were refixed, rewashed and acetylated for 10 min (197 ml H_2_O, 2.4 ml triethanolamine (Fluka 90279), 0.52 ml acetic anhydride), permeabilized for 15 min (1% Triton X-100 in PBS) and washed in PBS (3 × 5 min). Slides were then incubated for 2 h in 500 μl hybridization buffer (50% formamide, 5 × SSC, 5 × Denharts, 250 mg ml^−1^ bakers' yeast RNA (Sigma R6750) and 500 mg ml^−1^ herring sperm DNA) at RT in a 50% formamide/5 × SSC humidified chamber horizontal without coverslips. Hybridization solution was replaced with 75 μl fresh hybridization solution containing 200–400 ng ml^−1^ DIG probe that was heated to 80 °C for 5 min and iced. Slides were coverslipped and incubated in a 50% formamide/5 × SSC humidified chamber overnight at 70 °C. The next day, coverslips were removed by immersion of slides in 70 °C 5 × SSC, and slides were incubated in 0.2 × SSC at 70 °C for 1–3 h. Slides were then transferred to 0.2 × SSC for 5 min at RT, and incubated with 1–2 ml B1 (0.1 M Tris (pH 7.5) and 0.15 M NaCl) with 1% heat-inactivated goat serum (HINGS) for 1 h at RT. This was removed and 0.5 ml anti-DIG antibody (1:1000, Roche Cat# 11093274910) in B1+ HINGS was added prior to incubation overnight at 4 °C in a humidified chamber. Slides were subsequently washed with B1 (3 × 20 min) and equilibrated with B3 (0.1 M Tris (pH 9.5), 0.1 M NaCl and 50 mM MgCl_2_). B3 was removed and 200 μl B4 (75 mg ml^−1^ nitro-blue tetrazolium chloride (NBT), 50 mg ml^−1^ 5-bromo-4-chloro-3′-indolyphosphate p-toluidine salt (BCIP) and 0.24 mg ml^−1^ levamisole) was added to each slide and incubated up to 3 days in the dark until reactions were stopped in PBS. Slides were subsequently rinsed in water, air-dried and coverslipped in glycerol.

Antibodies used were as follows: mouse anti-HA antibody (12CA5, 1:1000, Roche, cat. no. 11666606001), rabbit anti-HA antibody (1:200, Sigma, cat. no. H6908), mouse anti-MPM2 (1:1,000, EMD Millipore, cat. no. 05-368MG), rabbit anti-Ki67 (1:1,000, Abcam, cat. no. ab15580), mouse anti-Lim1/2 (1:1, developmental studies hybridoma bank DHSB), 4F2), rabbit anti-Lhx3 (1:2,500, Abcam, cat. no. ab14555), rabbit anti-Foxp1 (1:10,000, from T.M. Jessell), rabbit anti-Er81 (1:32,000, from T.M. Jessell), guinea pig anti-Nkx6.1 (1:4,000, from T.M. Jessell); mouse anti-KDEL (10C3,1:200, Abcam, cat. no. ab12223), rabbit anti-Isl1/2(K5) (1:2,500, from T.M. Jessell), guinea pig anti-Isl1/2, (1:10,000, from T.M. Jessell); mouse anti-Isl2 (1:100, DSHB, 4H9); mouse anti-HB9/MNR2 (1:100, DSHB, 81.5C10); guinea pig anti-Olig2 (1:20,000, from B. Novitch), goat anti-β Gal (1:3,000, Arnel), rat anti-BrdU (1:100, Abcam, cat. no. ab6326), guinea pig anti-NeuroM (1:10,000, from B. Novitch) and rabbit anti-mPrdx4 (ref. 25; 1:10,000). Secondary antibodies (JacksonImmuno Research) were used according to the supplier's protocol.

TUNEL (terminal deoxynucleotidyl transferase dUTP nick end labeling) analysis utilized the ApopTag Red In Situ Apoptosis Detection Kit (Millipore S7165). Confocal micrographs were captured on a Zeiss LSM 5 PASCAL microscope. Bright-field images were captured on a Zeiss Axioskop2 microscope.

### Loss- and gain-of-function studies

For loss-of-function experiments, chick embryos were electroporated with shRNA and analysed at HH St19-21 (ref. 16). ShRNA target sequences were as follows:

Prdx4 shRNA1: 5′-AAGCTGACTTCCGTTTAATTA-3′

Prdx4 shRNA2: 5′-AAGGTCATTAATGGAGAGTTT-3′

control shRNA1: 5′-AATTCGCGCCTAGGTCCGAAC-3′

control shRNA2: 5′-AACTAGGTCGTTCGACGTAAG-3′

Sections (10–20) from each of five embryos with at least 80% loss of *Prdx4* mRNA expression were scored. Estimates of *Prdx4* mRNA loss were obtained by comparison of the area of *Prdx4* mRNA distribution visualized by *in situ* hybridization between the electroporated side of the spinal cord and the contralateral non-electroporated side (see panel in Fig. 2a for example of section designated as 80–85% *Prdx4* knockdown). Sections spanning the rostral–caudal axis were assayed for *Prdx4* expression in each embryo to evaluate *Prdx4* knockdown efficiency. For *Gde2*^*+/−*^ and *Prdx4* null mutant analysis, 10–20 sections from each of 6–8 E9.5 mouse embryos were counted. Cell cycle analyses were performed as follows^17^. BrdU (100 mg kg^−1^ body weight) was injected intraperitoneally into pregnant females 30 min and 16 h before embryo harvest to calculate S-phase and cell cycle exit indices, respectively. Sections were stained for BrdU, Ki67 and Mpm2, and the following was calculated: S-phase: BrdU^+^/Ki67^+^ cells, cell cycle exit: BrdU^+^Ki67^−^/BrdU^+^Ki67^+^ and M-phase: Mpm2^+^/Ki67^+^. Columnar counts of motor neurons span the rostro–caudal extent of the hindlimb identified by Isl2^+^Lhx1^+^ cells^18^. For gain-of-function experiments, chick embryos were labelled with BrdU for 30 min, 30 h after electroporation to delineate the VZ/IZ boundary^17^. Images of 10–20 sections per embryo from 4 to 10 embryos were scored. Sections were chosen to ensure equivalent electroporation efficiency by LacZ (GDE2) and HA (Prdx4) staining.

All work involving animals was performed in accordance with National Institutes of Health (NIH) regulations, and approved by Institutional Animal Care and Use Committees (IACUC) at the Johns Hopkins School of Medicine.

### Primary spinal motor neuron culture

Primary motor neuron cultures from spinal cords were prepared as follows^34^. Spinal cords from the brachial region were dissected from E11.5 mouse embryos in cold Hank's balanced salt solution (Invitrogen). Spinal cords were cut into small pieces and treated with dissociation solution containing l × Trypsin-EDTA (Gibco) and 0.2 mg ml^−1^ DNase I (Roche) for 15 min at 37 °C. Heat-inactivated horse serum (HIHS) and pre-culture medium (DMEM containing 10% fetal bovine serum (FBS)) were then added to stop the reaction. Cells were completely dissociated with gentle titration by 1 ml pipettes, centrifuged and resuspended with fresh pre-culture medium. Cells were then cultured in uncoated plastic dishes for 1 h at 37 °C to allow non-neuronal cells to adhere to the plastic dish. The neuron-enriched supernatant was collected and centrifuged, and the pellet was resuspended in MMTN medium (DMEM, 4% HIHS, 2% B27 (Gibco) and 1% N2 (Gibco) with fresh added growth factors (10 ng ml^−1^ glial cell line-derived neurotrophic factor (GDNF), brain-derived neurotrophic factor (BDNF), ciliary neurotrophic factor (CNTF) and neurotrophin-3 (NT3). Cells were then plated to poly-D-lysine (PDL)/laminin-coated glass chamber slides (Nalge Nunc International). After 2 days, cultured neurons were fixed in 4% paraformaldehyde (PFA) for 10 min at RT, washed with PBS and used for immunohistochemical analysis.

### Co-IP and western blot

Co-IP studies using FLAG-tagged GDE2 and HA-tagged Prdx4 forms in HEK293T cells were performed with GammaBind G sepharose (Amersham). Additional antibodies used (1 μg each) were as follows: rabbit anti-GDE2, mouse anti-Flag (M2, Sigma, cat. no. F3165) and mouse anti-Myc (9E10, DSHB). Western blotting utilized HA-horseradish peroxidase (HRP) (F7, 1:10,000, Santa Cruz, cat. no. sc-7392 HRP), rabbit anti-GDE2 (1:200,000) and rabbit anti-Prdx4 (1:1,000) antibodies. Co-IPs for GDE2 and Prdx4 complexes were performed using anti-FLAG M2 Affinity gel (Sigma); 100 mM maleimide was included to block free thiol groups and prevent thiol-disulfide exchange or thiol oxidation^35^. Protein complexes were eluted by non-reducing sample buffer and divided into two equal fractions, one of which was reduced with 10% β-mercaptoethanol (β-ME) before SDS–PAGE.

Uncropped versions of key western blots are included in Supplementary Figures 8–10.

### Surface biotinylation and biotin-labelling assays

Surface biotinylation assay was performed as follows^20^. Briefly, 40 h after transfection, cells were cooled on ice, washed twice with ice-cold PBS++ (PBS with 1 mM CaCl_2_ and 0.5 mM MgCl_2_, pH 7.4) and incubated with PBS++ containing 1 mg ml^−1^ Sulfo-NHS-SS-Biotin (Pierce) for 30 min at 4 °C. Unreacted biotin was quenched by PBS++ containing 100 mM glycine. Cells were lysed in RIPA buffer, sonicated and centrifuged for 20 min at 4 °C. The supernatant was incubated with neutravidin-agarose beads (Pierce) for 2 h at 4 °C. Biotinylated protein bound by neutravidin were washed with RIPA buffer and eluted with SDS sample buffer.

To detect disulfide bonds, biotin labelling was performed as previously described with modification^17^,^36^. Briefly, transfected cells were washed with cold PBS supplemented with 10 mM NEM (Sigma), lysed in 10 mM Tris (pH 7.0), 150 mM NaCl, 1% NP-40 and 0.1% SDS with 40 mM NEM and proteinase inhibitors at 4 °C for 30 min, and centrifuged. Supernatants were acetone precipitated and washed with 70% acetone, resolubilized in 4% SDS buffer (4% SDS, 50 mM Tris, 5 mM EDTA, pH 7.4) with 10 mM NEM with incubation at 37 °C for 10 min. Samples were then diluted with 3 volumes of LB buffer (150 mM NaCl, 50 mM Tris and 5 mM EDTA, pH 7.4) with 1 mM NEM, 0.2% Triton X-100 and proteinase inhibitor and incubated at 4 °C overnight with gently rocking. Next day, samples were acetone precipitated, washed and resolubilized in HENS buffer (250 mM HEPES (pH 7.7), 1 mM EDTA and 1% SDS) with 5 mM TCEP (tris (2-carboxyethyl) phosphine), and incubated for 1 h to reduce disulfide bonds. The reaction was quenched with acetone, and proteins were precipitated and washed as above. Pellets were resolubilized in HENS with 0.5 mM Biotin-HPDP (N-[6-(Biotinamido)hexyl]-3-(2-pyridyldithio)propionamide) (Soltec Ventures). Biotin labelling was carried out at RT for 90 min with rotating. The reaction was quenched again by acetone precipitation and the pellet was resolubilized in resuspension buffer (25 mM HEPES (pH 7.7), 0.1 mM EDTA and 0.75% SDS) and diluted with 6 volumes of binding buffer (1% Triton in TBS), then subjected to immunoprecipitation by anti-FLAG M2 Affinity gel (Sigma). Western blotting was performed using mouse anti-biotin antibody (1:10,000, Sigma, cat. no. B7653) and rabbit anti-GDE2 antibody (1:200,000).

GDE2 protein was similarly labelled for LC-MS/MS; however, 10 mM dithiothreitol was used instead of TCEP and 40 mM IAM was used instead of biotin-HPDP. GDE2 was immunoprecipitated by anti-FLAG M2 Affinity gel, resolved by SDS–PAGE and stained with Coomassie blue. Cysteines were identified by LC-MS/MS (Taplin Biological Mass Spectrometry Facility).

### Triton X-114 partitioning

Triton X-114 (Sigma) partitioning was performed as follows^20^. Triton X-114 (2%) was pre-conditioned in 100 mM Tris-HCl (pH 7.4) and 150 mM NaCl buffer. Cells were washed twice with PBS, incubated with 1% Triton X-114 buffer (10 min at 4 °C), and collected and iced for 10 min with occasional mixing. After centrifugation at 16,000*g* for 15 min at 4 °C, supernatants were diluted threefold with Triton X-114 buffer (final 1%). Two phases were separated by incubation at 30 °C for 5 min, followed by centrifugation at 3,000*g* for 3 min at RT. DT-rich pellet contained membrane-bound proteins, and DT-free supernatant was repeatedly extracted and analysed.

### Assays for motor neuron culture

Primary motor neuron culture was performed as described in the preceding section, except that the dissected spinal cords were treated with dissociation solution containing l × Accumax (Sigma) and 0.2 mg ml^−1^ DNase I (Roche) for 15 min at 37 °C. HIHS and DMEM medium (containing 10% FBS) were then added to stop the reaction. Cells were completely dissociated with gentle titration, centrifuged and resuspended with fresh MMTN medium. Cells were then plated onto poly-L-lysine (PLL) (Sigma, 0.2 mg ml^−1^) plus laminin (Sigma, 0.01 mg ml^−1^)-coated six-well plates. The density for plating is ∼1 embryo per well. Culture medium was changed 2 h after plating and half changed every 2 days.

At (days *in vitro*) DIV5, 200 μM H_2_O_2_ was added to the medium for 30 min, repeated four times in 2 h before harvesting cells. For ROS scavenger treatment, 50 μM Edaravone or 200 μM Tempol were added to growth medium for 24 h before harvesting cells for surface biotylation^31^. Surface biotinylation was performed as described in the preceding section^20^, except after labelling with Sulfo-NHS-SS-Biotin, cells were lysed in 1% Triton X-114 buffer with 100 mM maleimide and subjected to partitioning. DT-rich pellet were resuspended in RIPA buffer without 1% Triton X-100 and then incubated with neutravidin-agarose beads (Pierce) for 2 h at 4 °C. Biotinylated protein bound by neutravidin was washed with RIPA buffer and eluted with SDS sample buffer. Western blotting was performed using rabbit anti-mGDE2 antibody (1:4,000), rabbit anti-mPrdx4 antibody (1:1,000) and mouse anti-actin antibody (C4, 1:5,000, Millipore, cat. no. MAB1501).

### Live-cell staining

HEK293 cells were transfected with mGDE2FLAG and HAPrdx4 constructs. After 24 h, the culture medium was changed to serum-free DMEM. Forty to forty-eight hours after transfection, cells were pre-incubated with 5 μg ml^−1^ of chlorpromazine in Hank's balanced salt solution for 30 min at 4 °C, followed by deglycosylation with PNGase F at 37 °C for 1 h. After washing with PBS, cells were incubated with rabbit anti-mGDE2 loop antibody (1:2,000) in DMEM plus 10% FBS at 37 °C for 1 h, followed by secondary antibody incubation at RT for 45 min. The surface staining was fixed with 4% paraformaldehyde for 10 min before permeabilization and staining with FLAG and HA antibodies.

### Statistical analyses

All statistical tests utilized two-tailed, unpaired Student's *t*-test. All data are shown as mean±s.e.m. A value of *P*<0.05 was considered significant. The numbers analysed in each experiment (*n*) are either derived from individual experiments or individual embryos as stated in the figure legends. Analysis of embryos utilized ∼10–20 sections for each embryo.

## Author contributions

Y.Y. performed all the experiments in the paper and was assisted in the analysis of mouse phenotypes and cell counts by C.W. J.F provided *Prdx4* null animals. Y.Y. and S.S conceived ideas, designed experiments and analysed data; S.S. wrote the manuscript; and all authors reviewed and approved the manuscript.

## Additional information

**How to cite this article:** Yan, Y. *et al.* Prdx4 is a compartment-specific H_2_O_2_ sensor that regulates neurogenesis by controlling surface expression of GDE2. *Nat. Commun.* 6:7006 doi: 10.1038/ncomms8006 (2015).

## Supplementary Material

Supplementary Information Supplementary Figures 1-10.
