# Supplementary material for: Prdx4 is a compartment-specific H2O2 sensor that regulates neurogenesis by controlling surface expression of GDE2
Source: Nat Commun. 2015 May 6;6:7006. doi: 10.1038/ncomms8006 (PMC4432624; doi:10.1038/ncomms8006)
Supplement: Supplementary Information — Supplementary Figures 1-10. [file ncomms8006-s1.pdf]

Supplementary information

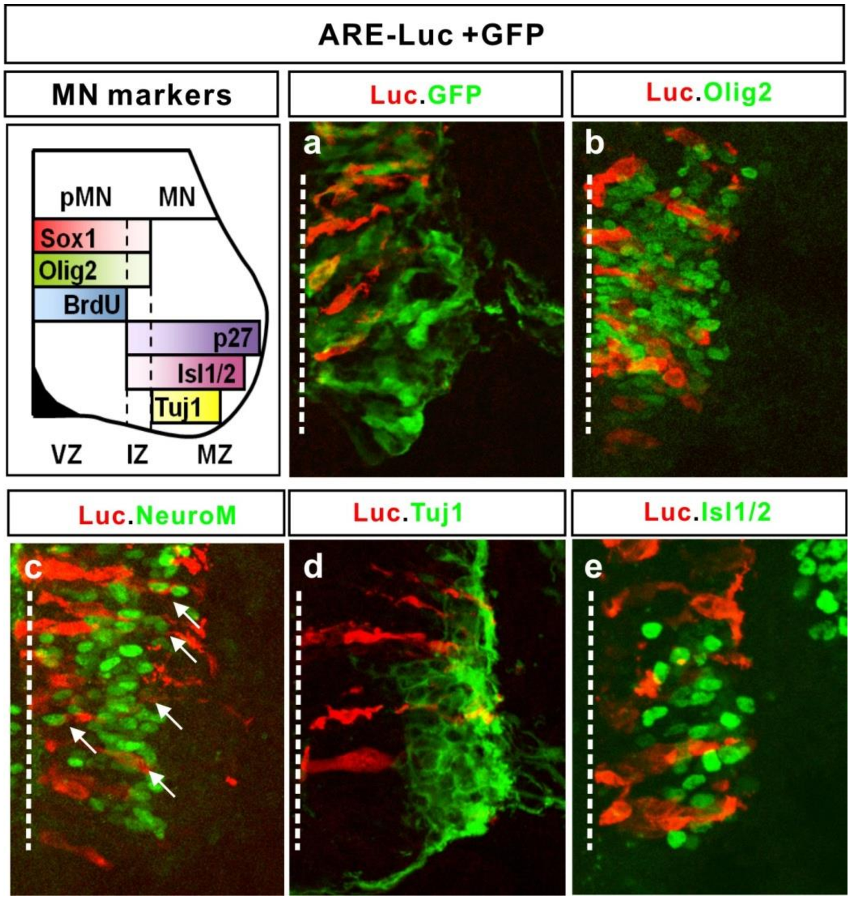

Supplementary Figure 1. VZ and IZ cells have higher oxidative levels than neurons in the MZ.

(Left) Schematic of lower right quadrant of the spinal cord delineating the motor neuron progenitor domain (pMN). Motor neuron progenitors are identified by Sox1 and Olig2 expression, and BrdU incorporating cells mark the lateral boundary of the ventricular zone (VZ). As cells differentiate, the cell bodies move into the intermediate zone (IZ) and express low levels of progenitor and postmitotic markers. Fully differentiated motor neurons (MN) reside in the marginal zone (MZ) and are identified by coexpression of p27, Isl1/2 and Tuj1. (**a-e**) Sections of electroporated chick spinal cords electroporated on the right with ARE-luciferase reporter constructs. Dotted lines mark the central midline. (**a**) Coelectroporation with GFP tracer plasmids shows efficiency of electroporation. Luciferase is expressed in Olig2$^+$ progenitors in the VZ (**b**) and in some NeuroM$^+$ cells in the IZ (arrows, **c**) but not in postmitotic motor neurons in the MZ (**d, e**).

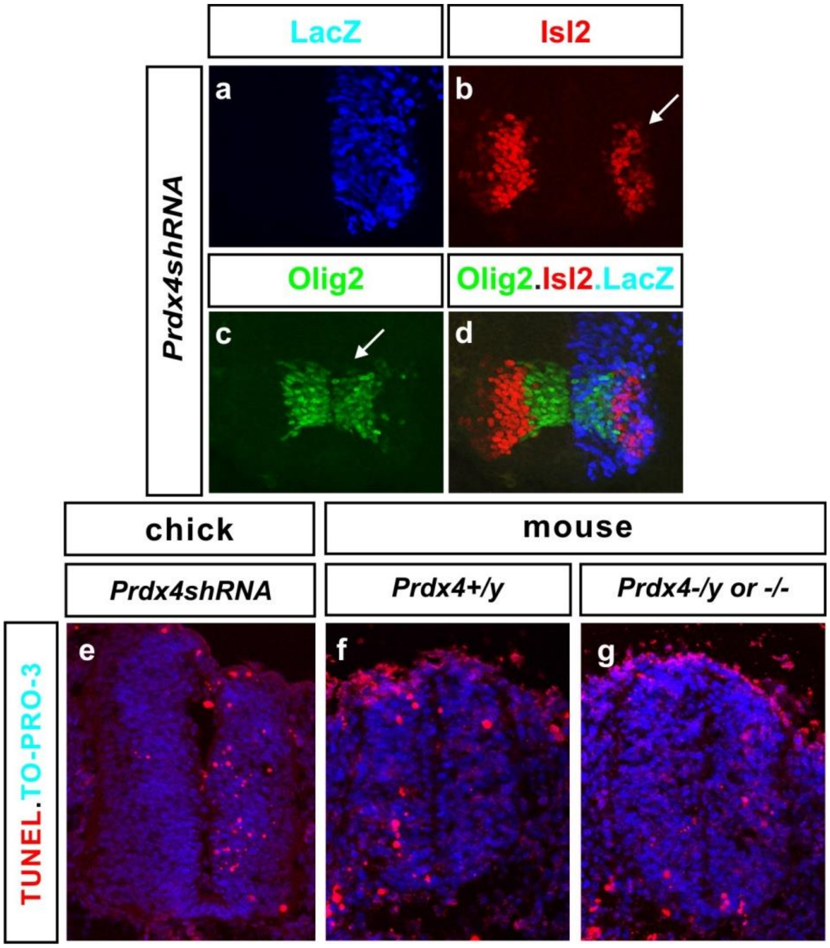

Supplementary Figure 2. Loss of Isl^{+} motor neurons in *Prdx4* shRNA embryos is accompanied by cell death.

(**a-e**) Sections of chick embryonic spinal cords electroporated with *Prdx4* shRNAs on the right. Arrow in (**b**) indicates loss of Isl2^{+} motor neurons. Arrow in (**c**) shows decreased Olig2 expression. TUNEL analysis in (**e**) shows increased cell death when Prdx4 is ablated. (**f, g**) Sections of mouse spinal cords at E9.5 show no changes in cell death as visualized by TUNEL in *Prdx4* null embryos.

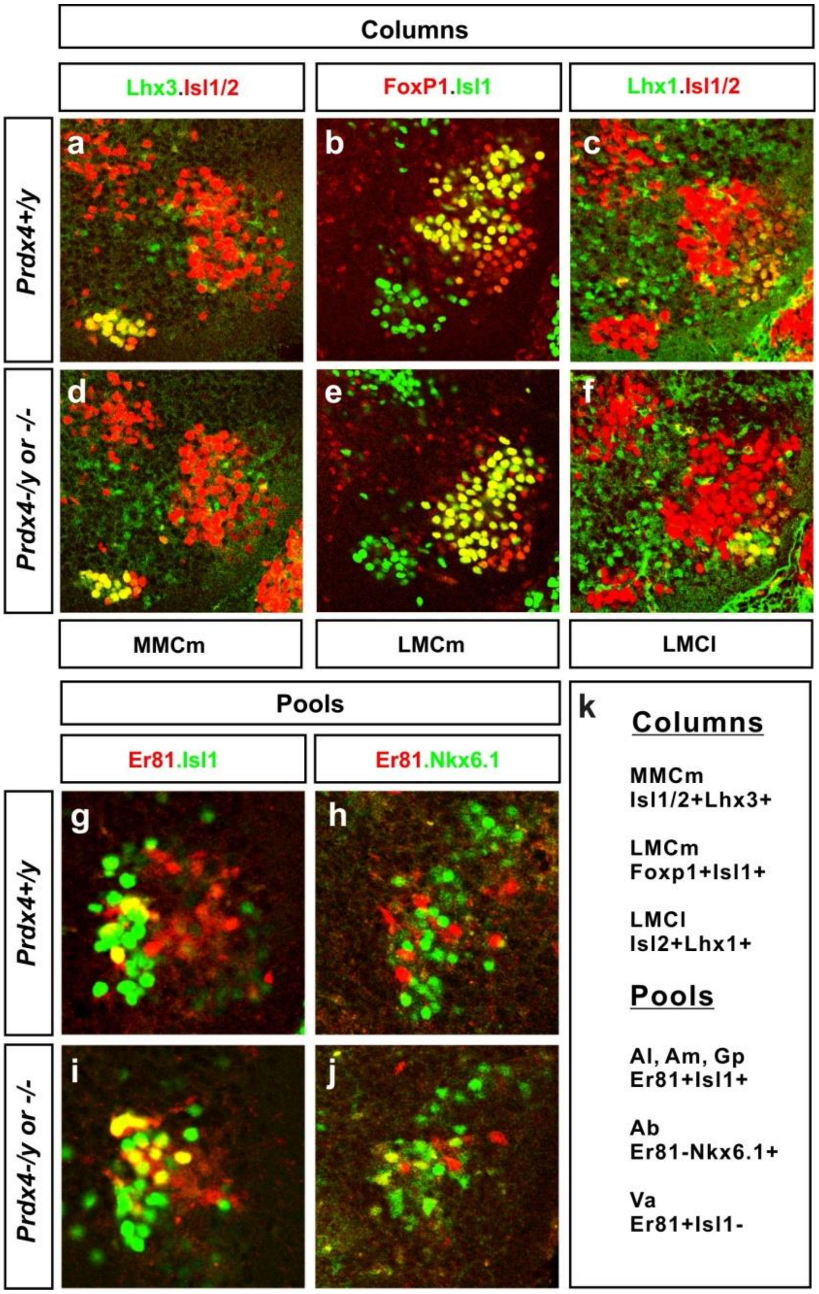

Supplementary Figure 3. Early born motor columns and pools are expanded in the absence of Prdx4.

(**a-j**) Confocal images of the ventral right quadrant of sectioned E13.5 mouse spinal cords stained with antibodies to identify hindlimb motor columns and LS2 motor pools. (**k**) List of markers used to molecularly identify MMCm, LMCm and LMCl motor columns; and adductor longus (Al), adductor magnus(Am), gracilis posterior (Gp), adductor brevis (Ab) and vasti (Va) motor pools.

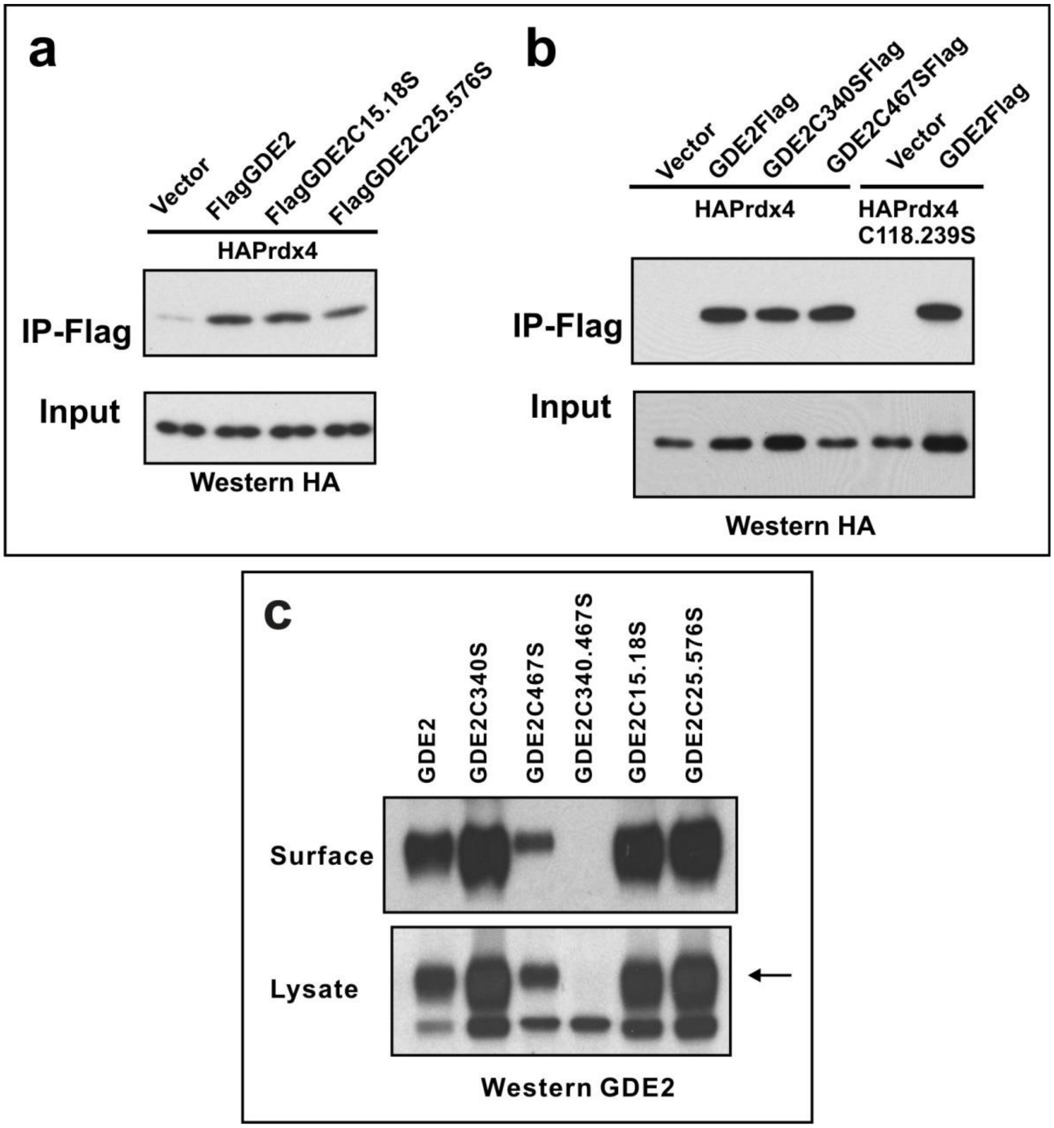

Supplementary Figure 4. Majority of GDE2 mutants are expressed on the cell surface, and can interact with Prdx4.

(**a, b**) Western blots of coIPs of Prdx4 and GDE2 show that WT and redox inactive Prdx4 both interact with GDE2, and that Prdx4 interacts with mutant forms of GDE2. (**c**) Western blot of surface biotinylation assays performed in transfected HEK293T cells. All GDE2 mutants with the exception of GDE2C340.467S show appropriate glycosylation patterns (arrow) and surface localization.

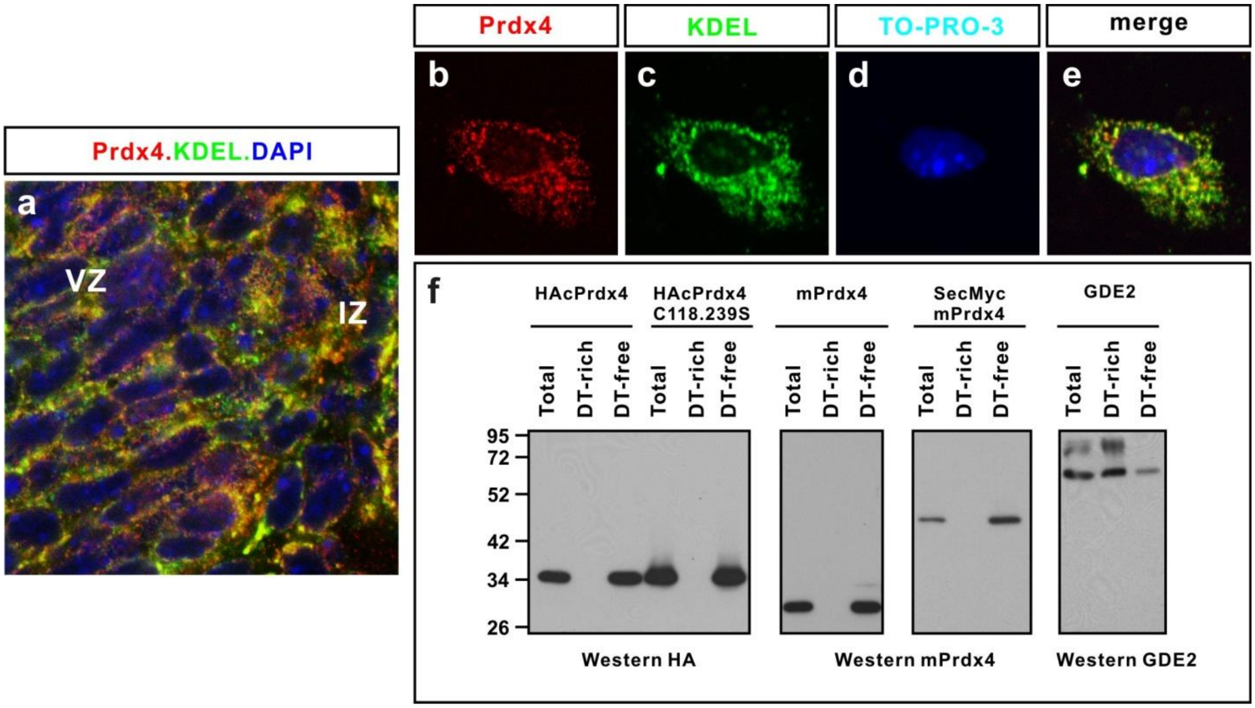

Supplementary Figure 5. Prdx4 is localized to the ER lumen.

(a) Section of mouse embryonic spinal cord showing colocalization of Prdx4 (red) with the ER marker KDEL (green) in VZ progenitors and in newly differentiating motor neurons in the IZ. (**b-e**) Example of primary motor neuron cultured in vitro stained with Prdx4, KDEL and To-Pro-3 to identify nuclei. Prdx4 overlaps with KDEL expression suggesting that Prdx4 is localized to the ER. (**f**) Western blot of Triton X114 partitioning of extracts of transfected HEK293T cells. HA-tagged Prdx4 and redox inactive Prdx4C118.239S, mouse Prdx4 (mPrdx4), and Prdx4 tagged with sequences that target proteins to the secretory pathway (SecmycmPrdx4), all partition to DT-free fractions, indicating that they are located within the ER lumen. In contrast, glycosylated forms of GDE2 partition to DT-rich fractions while non/hypoglycosylated GDE2 partitions to DT-free fractions.

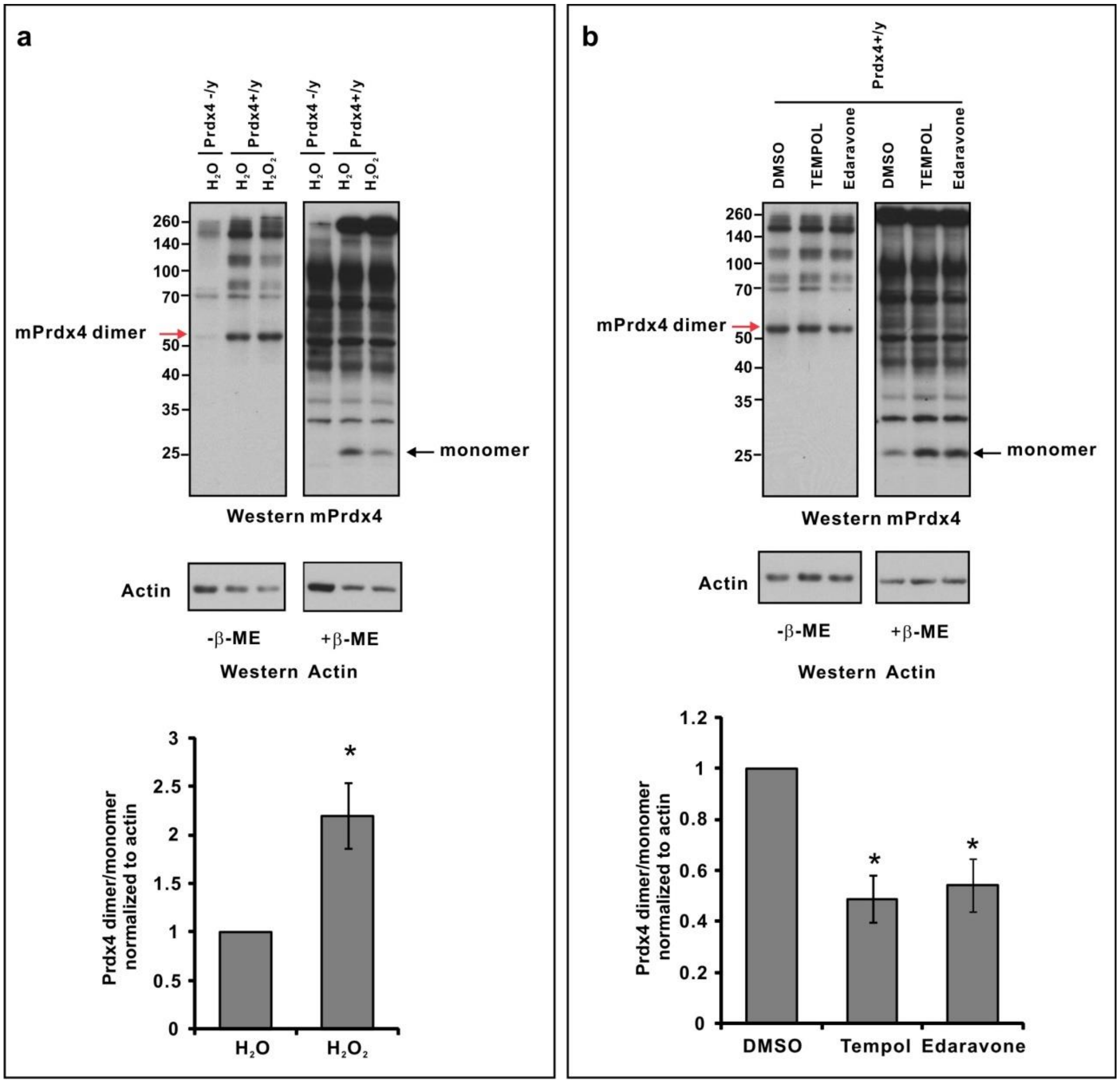

Supplementary Figure 6. Prdx4 dimer formation in primary motor neuron extracts is affected by cellular redox.

(**a, b**) Western blots of DT-free fractions of Triton X-114 extractions of primary motor neuron cultures derived from E11.5 spinal cords grown in the presence of H_2O_2, or the ROS scavengers Tempol or Edaravone. Red arrow marks Prdx4 dimers detected in the absence of the reducing agent β-mercaptoethanol (β-ME); black arrow marks total amounts of Prdx4 monomers generated by incubation with β-ME. Bands specific to Prdx4 are identified as absent in Prdx4 knockouts (*Prdx4*$^{-/y}$). Graphs show ratios of Prdx4 dimers to monomers after normalization to actin. Mean ± SEM; (**a**) *p=0.028, n=9 separate cultures; (**b**) Tempol *p=0.005, Edaravone *p=0.011, n=5 separate cultures, two-tailed student's t-test.

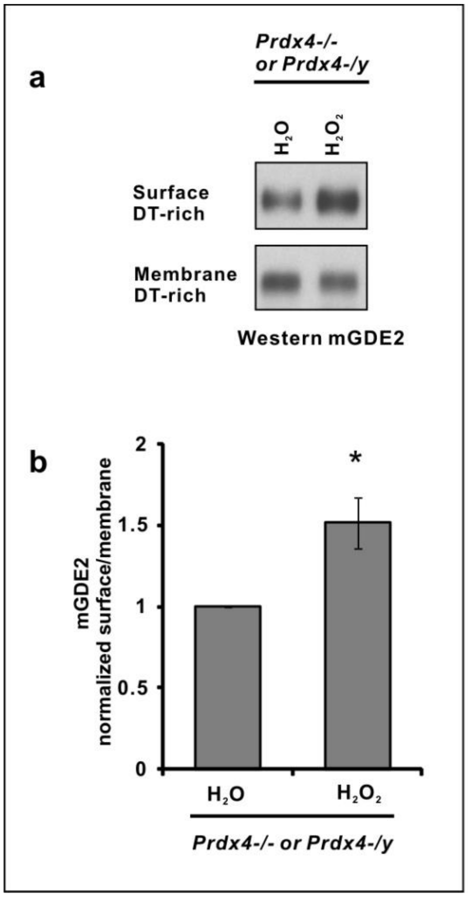

Supplementary Figure 7. GDE2 surface expression is elevated by H_2O_2 in the absence of Prdx4

(**a**) Representative Western blot of DT-rich fractions of Triton X-114 extractions of primary motor neuron cultures derived from E11.5 spinal cords of *Prdx4* null animals grown in the presence of H_2O_2, or H_2O. (**b**) Graph quantifying GDE2 surface expression in **a**. Mean $\pm$ SEM; n=15 embryos, *p= 0.0033, two-tailed student's t-test.

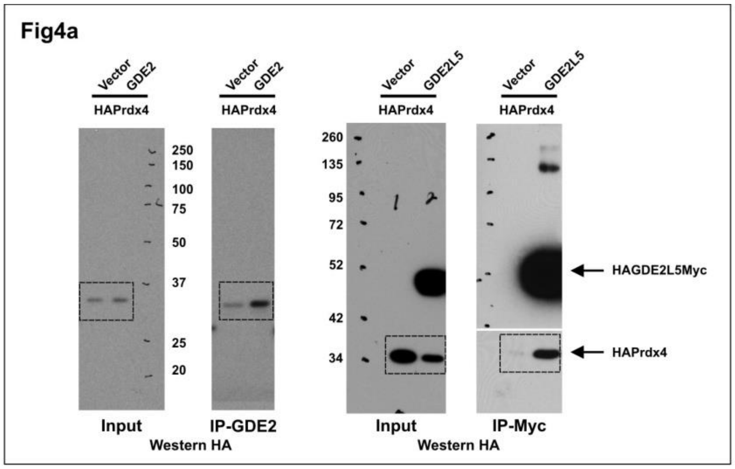

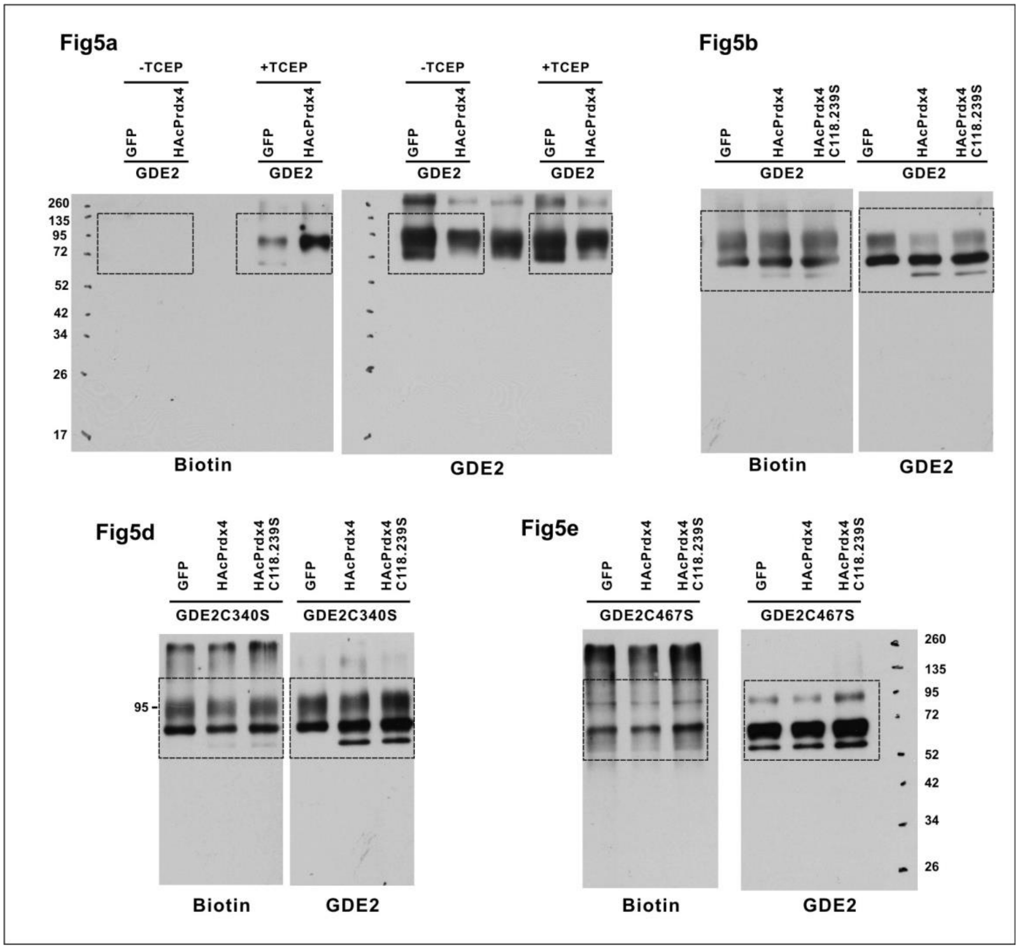

Supplementary Figure 8. Western blots for Figures 4 and 5.

Uncropped images of Western blots for Figure 4a, Figure 5a, Figure 5b, Figure 5d and Figure 5e in the main text. Boxes in each panel correspond to the cropped images shown in the main text.

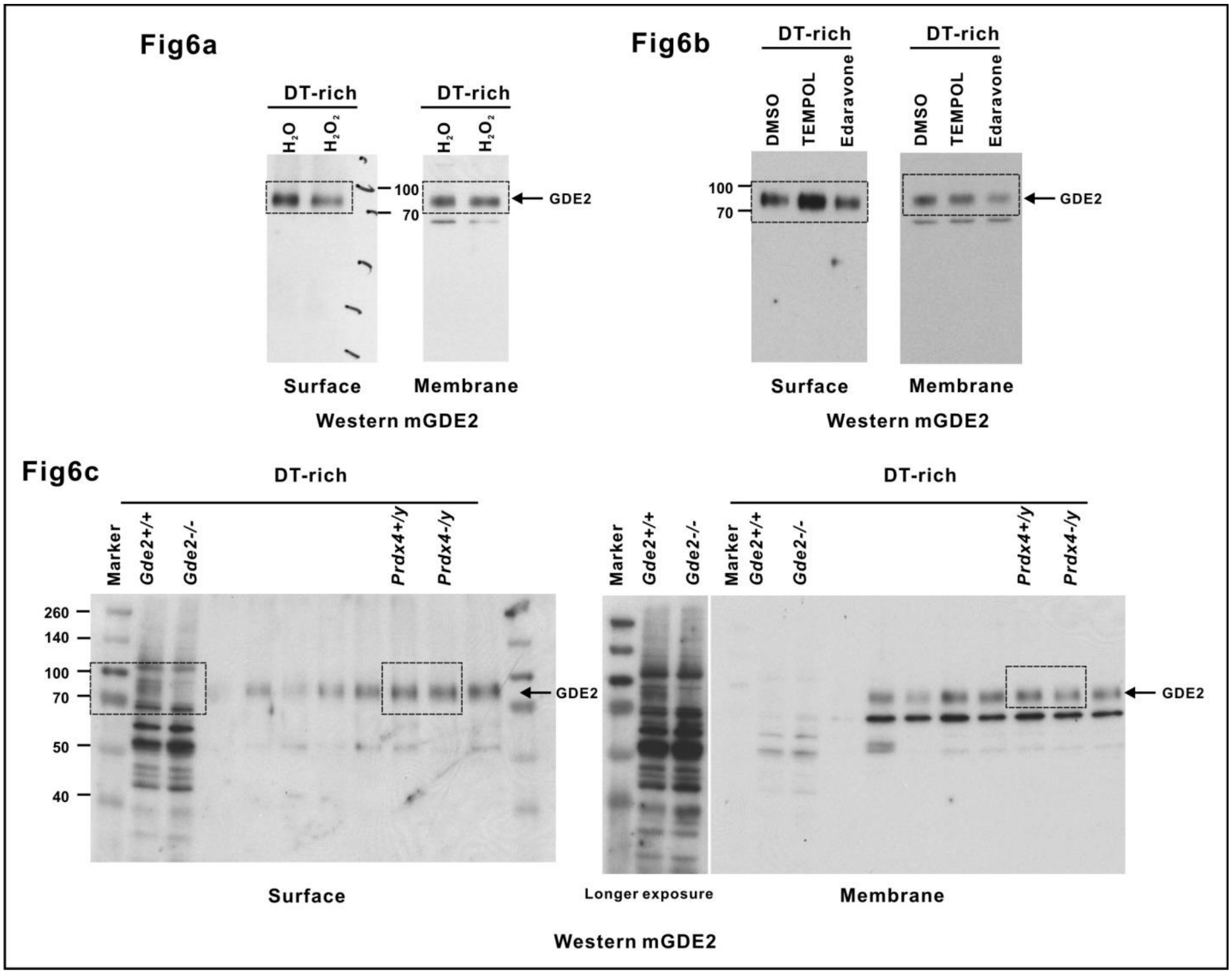

Supplementary Figure 9. Western blots for Figure 6.

Uncropped images of Western blots for Figure 6 in the main text. Boxes in each panel correspond to the cropped images shown in the main text.

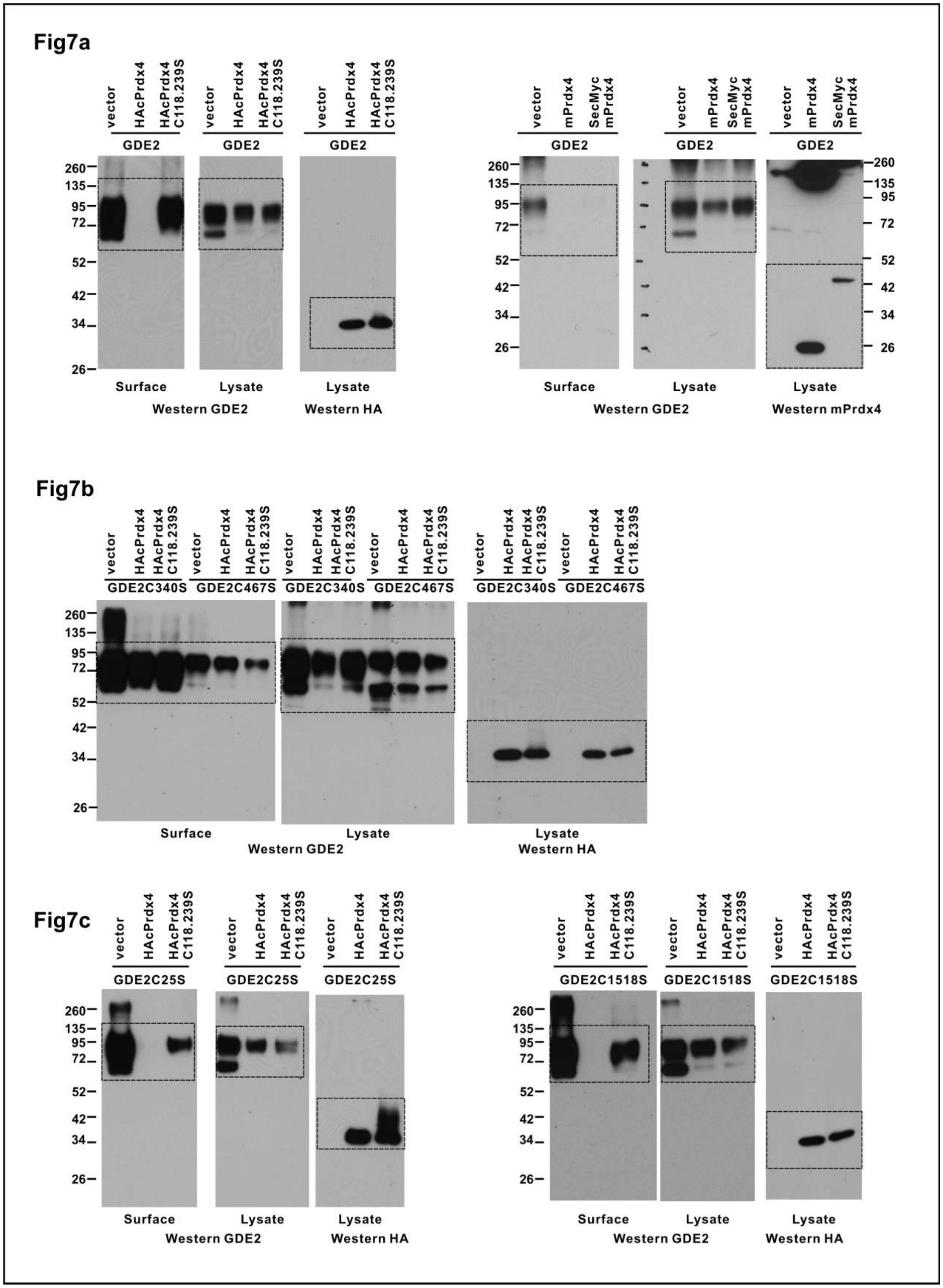

Supplementary Figure 10. Western blots for Figure 7.

Uncropped images of Western blots for Figure 7 in the main text. Boxes in each panel correspond to the cropped images shown within panels of figures in the main text.
